# Integrative taxonomy refutes a species hypothesis: The asymmetric hybrid origin of *Arsapnia arapahoe* (Plecoptera, Capniidae)

**DOI:** 10.1002/ece3.4852

**Published:** 2019-01-13

**Authors:** Michael K. Young, Rebecca J. Smith, Kristine L. Pilgrim, Matthew P. Fairchild, Michael K. Schwartz

**Affiliations:** ^1^ U.S. Forest Service, Rocky Mountain Research Station, National Genomics Center for Wildlife and Fish Conservation Missoula Montana; ^2^ U.S. Forest Service, Arapaho and Roosevelt National Forest Fort Collins Colorado

**Keywords:** cryptic taxa, ESA, GBS, hybrid zone, nonintrogressive hybridization, stonefly

## Abstract

Molecular tools are commonly directed at refining taxonomies and the species that constitute their fundamental units. This has been especially insightful for groups for which species hypotheses are ambiguous and have largely been based on morphological differences between certain life stages or sexes, and has added importance when taxa are a focus of conservation efforts. Here, we examine the taxonomic status of *Arsapnia arapahoe*, a winter stonefly in the family Capniidae that is a species of conservation concern because of its limited abundance and restricted range in northern Colorado, USA. Phylogenetic analyses of sequences of mitochondrial and nuclear genes of this and other capniid stoneflies from this region and elsewhere in western North America indicated extensive haplotype sharing, limited genetic differences, and a lack of reciprocal monophyly between *A. arapahoe* and the sympatric *A. decepta*, despite distinctive and consistent morphological differences in the sexual apparatus of males of both species. Analyses of autosomal and sex‐linked single nucleotide polymorphisms detected using genotyping by sequencing indicated that all individuals of *A. arapahoe* consisted of F_1_ hybrids between female *A. decepta* and males of another sympatric stonefly, *Capnia gracilaria*. Rather than constitute a self‐sustaining evolutionary lineage, *A. arapahoe* appears to represent the product of nonintrogressive hybridization in the limited area of syntopy between two widely distributed taxa. This offers a cautionary tale for taxonomists and conservation biologists working on the less‐studied components of the global fauna.

## 1 INTRODUCTION

Within biology, arguably the most fundamental undertaking is that of defining a species. Proposals on what constitutes a species are legion; although the particulars vary depending on which of the many forms of evidence are used to delineate them—morphological, genetic, ecological, reproductive, geographical, or some combination—there is consensus that species constitute independently evolving lineages to which are assigned names (De Queiroz, 2007). Because all species are hypotheses, a species name is no more than the label for a particular hypothesis. But given the central role of species to biology, these names profoundly influence how we think about the elements and conservation of biodiversity.

Although most taxonomies of organisms are based on morphological characters, genetic tools are essential to refining these taxonomies by parsing genotypic variation across demographic, geographic, and taxonomic scales, discerning recent and ancient introgression, revealing cryptic taxa, and synonymizing dubious ones (Kjer, Simon, Yavorskaya, & Beutel, 2016). Perhaps most straightforward has been the genetic assignment of specimens to known lineages by linking their genotypes to those of named and phenotypically identified voucher specimens. This is the province of DNA barcoding—assigning individuals to species based on their genetic sequences, generally of the cytochrome c oxidase subunit 1 (COI) mitochondrial region—and it has been applied to taxa across all of life, with an emphasis on animals (Hebert, Cywinska, & Ball, 2003). One of the first demonstrations of its efficacy involved insects (Hebert, Penton, Burns, Janzen, & Hallwachs, 2004), and they have been the focus of initiatives to associate DNA barcodes with individual species across higher taxonomic categories and throughout particular geographies (Smith et al., 2008; Zhou et al., 2016). These efforts have been broadly successful (Blagoev et al., 2016; Webb et al., 2012), in part because they permit linking morphologically cryptic larvae or females with their more easily recognized adult male counterparts (Gamboa & Monaghan, 2014). For some groups of insects, however, these broad assessments are in their infancy and concordance between morphological and genetic identifications of forms has been uneven (Geiger et al., 2016). In part, this reflects taxonomies erected on morphological grounds that have not yet been evaluated from a molecular perspective (Schlick‐Steiner et al., 2010). This pattern also derives, however, from discord in the phylogenetic signal among different genes, especially for taxa of recent origin or exposed to introgressive hybridization (Funk & Omland, 2003). Taxonomic instability is expected given that all names are hypotheses about evolutionary lineages, and their revision is straightforward, albeit nontrivial, from a scientific perspective (Valdecasas, Williams, & Wheeler, 2008). But getting the names right is more than an academic exercise; society makes outsize investments in some of these hypotheses, particularly when they are associated with at‐risk taxa that are the focus of conservation (Mace, 2004; Schwartz & Boness, 2017).

An exemplar of many these issues is the Arapahoe snowfly, *Arsapnia arapahoe* Nelson & Kondratieff (Plecoptera: Capniidae), one of eight species of stoneflies in the western North American genus *Arsapnia* that were formerly assigned to the *Capnia decepta* Banks group (Murányi, Gamboa, & Orci, 2014; Nelson & Baumann, 1989). This species was originally described from single male specimens collected in two streams in 1986 and 1987 in the Cache la Poudre River basin in north‐central Colorado (Nelson & Kondratieff, 1988), and not observed again in these streams until March 2009, when the first putative females were also collected (Heinold & Kondratieff, 2010). More recent surveys for adults extended this distribution to 19 additional sites as far south as the South Platte River basin in the Colorado Front Range (Fairchild, Belcher, Zuellig, Vieira, & Kondratieff, 2017); larval forms remain unknown. Where present, this species was outnumbered by orders of magnitude by two sympatric capniids, *A. decepta* and *Capnia gracilaria* Claassen (Fairchild et al., 2017; Heinold, Gill, & Belcher, 2014). Its restricted range and apparent rarity led to a petition and subsequent candidacy for its listing under the U.S. Endangered Species Act (U.S. Fish and Wildlife Service, 2012).

The life history, morphology, and systematics of capniid stoneflies, however, make assessing the conservation status of many species, including *A. arapahoe*, a challenge. Members of this family tend to emerge as adults in late winter and early spring and can be locally abundant (Baumann, Gaufin, & Surdick, 1977), but the mating period is brief and synchronized at any particular location. Larvae apparently occupy hyporheic habitats that make them difficult to capture in benthic surveys, and this life stage can rarely be identified to species (Stewart & Stark, 1988). This is also true of adult females, for example, differences between *A. arapahoe* and *A. decepta* are thought to be subtle at best (Heinold & Kondratieff, 2010), and females of different species in this genus may be easily mistaken for one another and for females in confamilial taxa (Nelson & Baumann, 1989). Even male identification can be problematic. The shape, size, and ornamentation of the male reproductive organ often constitutes the basis for describing and identifying species, yet this structure can exhibit substantial local and range‐wide variation within taxa (Baumann & Stark, 2017). Male *A. arapahoe*, however, are readily identifiable because the epiproct lacks the mesal bulbous projections typical of all other members of this genus (Nelson & Kondratieff, 1988). This characteristic is so distinctive that it led Nelson and Kondratieff (1988) to speculate that *A. arapahoe* was the sister taxon to all other members of *Arsapnia*, and perhaps most closely related to *A. sequoia* Nelson & Baumann and *A. utahensis* Gaufin & Jewett, not the sympatric *A. decepta*. The position of these species within Capniidae is also ambiguous; systematists have long regarded Capniidae as a synthetic, paraphyletic assemblage in need of revision (Murányi et al., 2014; Nelson & Baumann, 1989).

Confusion about the taxonomic position of *A. arapahoe* grew when an attempt to use genetic tools to identify it (Heinold, Gill, Belcher, & Verdone, 2014) produced unexpected results: The interspecific distance between sympatric male *A. arapahoe* and *A. decepta* (0.10%) was typical of variation found within species of stoneflies (0.35%–0.53%; Gill et al., 2013; Morinière et al., 2017; Zhou, Jacobus, DeWalt, Adamowicz, & Hebert, 2010, but see Gill, Sandberg, & Kondratieff, 2015 for much higher estimates), not between species (generally ≥2%; Zhou, Adamowicz, Jacobus, DeWalt, & Hebert, 2009). Paradoxically, a putative female allotype of *A. arapahoe* shared the haplotype of a distantly related species in a different genus, *Capnura wanica*. The incidence of interspecific haplotype sharing and low interspecific divergence is rare in many arthropods (e.g., <2% among Canadian spiders (Blagoev et al., 2016) and virtually nonexistent in North American mayflies (Webb et al., 2012)), making assignment of different sexes to different species highly unusual and difficult to ascribe to a single mechanism. More likely is that a combination of factors is responsible, among them misidentification of voucher specimens, incomplete lineage sorting between recently diverged species, interspecific haplotype sharing caused by infection by the bacterium *Wolbachia*, or hybridization (Funk & Omland, 2003; Smith et al., 2012). Heinold et al. (2014) favored incomplete lineage sorting, arguing that the dramatic morphological differences between males were reliable and obvious evidence of speciation, haplotypes were not those of the markedly divergent *Wolbachia* sequences (and selective sweeps driven by *Wolbachia* infection are extraordinarily rare; Smith et al., 2012), and hybridization was not likely because of the lack of morphological intermediates between male *A. arapahoe* and *A. decepta*. But this interpretation did not account for the differences among male and female genotypes and failed to satisfy the requirement that an integrative taxonomy provides an evolutionary explanation for all aspects of morphological and molecular discord (Schlick‐Steiner et al., 2010).

A simple explanation for haplotype sharing among these taxa would be hybridization, but this phenomenon has been regarded as rare among stoneflies and other aquatic arthropods (Dijkstra, Monaghan, & Pauls, 2014; Hughes, Finn, Monaghan, Schultheis, & Sweeney, 2014), and until recently was thought to be confined to a few species pairs of eastern North American *Allocapnia* (Ross & Ricker, 1971). That hybridization might be more prevalent is discouraged by the biological species concept with its predisposition to view hybridization as a rare accident (Mallet, 2005) and by notions that behavioral or anatomical differences constitute intrinsic reproductive barriers. For example, conspecific drumming signals used by male and female stoneflies for mate recognition are regarded as species‐specific and likely to ensure pre‐zygotic reproductive isolation (Boumans & Johnsen, 2014; Stewart, 2001). Nonetheless, there are many examples of arthropod taxa with elaborate pre‐mating and putatively species‐specific displays that nonetheless result in attempted mating between species (Masly, 2012), including between stoneflies in different genera (Zeigler, 1990). Even if hybridization does not ensue, assuming species identity based on temporary male–female association could lead to misidentification of the less morphologically distinct females. There may be a stronger argument for the rarity of hybridization based on the “lock and key” hypothesis (Sota & Kubota, 1998), which posits that anatomical complementarity of male and female terminalia is required for successful reproduction, but again there are a host of examples demonstrating hybridization between anatomically disparate taxa (Shapiro & Porter, 1989). Nonetheless, the success of heterospecific crosses may be asymmetric because of pre‐ or post‐zygotic reproductive incompatibilities, that is, crosses between a male of one species and a female of the other may exhibit lower female survival, likelihood of insemination, or fitness of offspring than does the opposite pairing (Masly, 2012).

Our goal was to resolve the taxonomic ambiguity surrounding *A. arapahoe* via an iterative approach to integrative taxonomy (Yeates et al., 2011). We treated the morphological identifications as authoritative hypotheses to be evaluated in light of molecular data from *A. arapahoe* and related capniid stoneflies. To that end, we analyzed sequences of two mitochondrial regions and one nuclear gene. Because these results were inconclusive, we used genotyping by sequencing to more thoroughly explore the evolutionary origin and taxonomic validity of *A. arapahoe*.

## 2 MATERIALS AND METHODS

### 2.1 Sample collection and sequence selection

**Figure 1:**
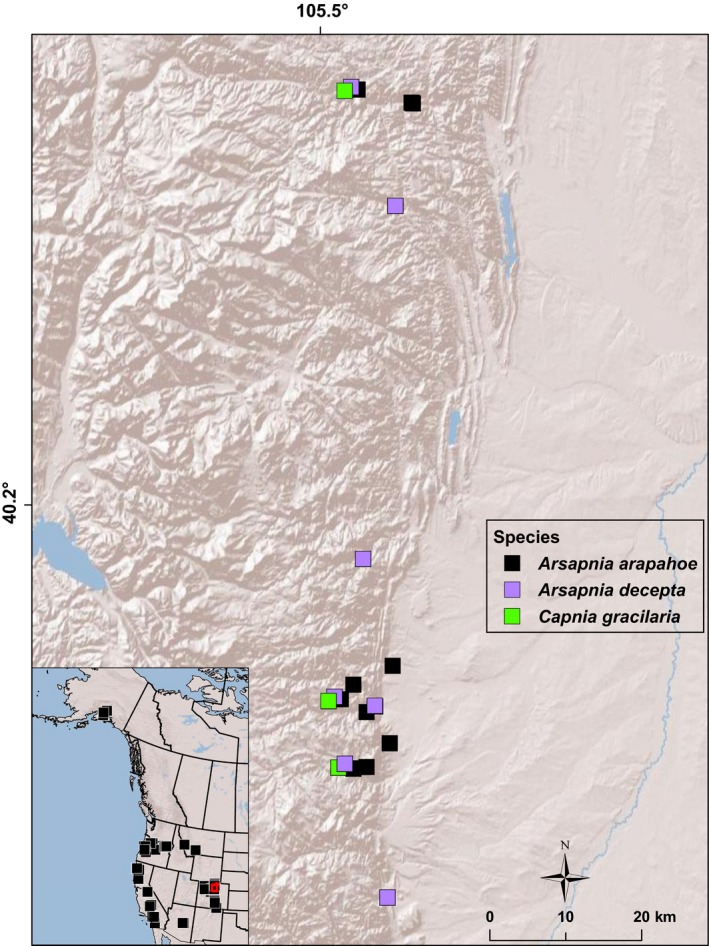
Locations of all specimens of capniid stoneflies examined in this study (lower left) and along the northern Colorado (USA) Front Range (inset area in red, expanded)

Specimens for sequencing were collected for this study or drawn from the collections at the C.P. Gillette Museum of Arthropod Diversity, Colorado State University, Fort Collins, Colorado (Figure 1; Supporting Information Table S1). All individuals were identified to species by taxonomic experts at this facility. Furthermore, we re‐examined every specimen under a dissecting microscope to confirm the sex of the individuals being genetically sequenced, and of all *A. arapahoe* specimens to ensure they were of this species. We examined specimens of: (a) *A. arapahoe* from across its northern Colorado range; (b) *A. decepta* from northern Colorado and Arizona; (c) additional members of the genus *Arsapnia*; and (d) other capniids from the range of *A. arapahoe* (e.g., *Capnia gracilaria*, *Capnura wanica* Frison) and elsewhere in western North America. These samples were supplemented with sequences of other specimens from these groups that were available in public databases (Supporting Information Table S2). There are two caveats. First, not all members of all groups were used in every analysis because of cost and because genotyping was not universally successful. Second, because our phylogenetic analyses (see below) revealed that polytomies were evident throughout the broad suite of species in different genera of Capniidae, we were uncertain as to which species might be relevant to understanding the phylogenetic identity of *A. arapahoe*. To that end, our phylogenetic analyses using two mitochondrial genes and one nuclear gene (Supporting Information Figures S2 and S3) of western North American capniid stoneflies identified a monophyletic clade that consisted of all species of the genus *Arsapnia* (*A. arapahoe*, *A. coyote *Nelson & Baumann, *A. decepta*, *A. pileata* Jewett, *A. sequoia*, *A. teresa* Claassen, *A. tumida* Claassen, and *A. utahensis*), two species of *Sierracapnia* (*S. barberi* Claassen and *S. palomar* Nelson & Baumann 1987), and three species of *Capnia* (*C. elongata* Claassen, *C. gracilaria*, and *C. promota* Frison), and we restrict presentation of our results to these taxa (hereafter, *Arsapnia* group), except where references to additional taxa are pertinent.

### 2.2 DNA sequencing

We used the QIAGEN DNeasy Blood and Tissue kit (QIAGEN Inc.) to extract genomic DNA from whole hind legs or the thorax of individual specimens, following the manufacturer's instructions for tissue. We sequenced COI and cytochrome b (cyt *b*) to facilitate comparisons with sequences in existing databases (Ratnasingham & Hebert, 2007) and to increase the taxonomic resolution of the mitochondrial data (Hillis, Pollock, McGuire, & Zwickl, 2003). We sequenced the ribosomal internal transcribed spacer (ITS1) to permit comparison of a nuclear phylogeny to those from the mitochondrial genes and to assess whether hybridization or sharing of mitochondrial genes associated with *Wolbachia* infection were evident. We amplified COI using the standard primers (LCO1490/HCO2198 (Folmer, Black, Hoeh, Lutz, & Vrijenhoek, 1994) or LepF1/LepR1 (Hebert et al., 2004)), cyt *b* using primers developed by Jordan et al. (2016), and ITS1 using primers developed by Pilgrim, Roush, and Krane (2002). We used reaction volumes of 50 μl containing 50–100 ng DNA, 1 × reaction buffer (Life Technologies), 2.5 mm MgCl_2_, 200 µm each dNTP, 1 µm each primer, and 1 U Taq polymerase (Life Technologies). The PCR program was 94°C/5 min, [94°C/1 min, 55°C/1 min, 72°C/1 min 30 s] × 34 cycles, and 72°C/5 min. The quality and quantity of template DNA were determined by 1.6% agarose gel electrophoresis. Products from PCR were purified with Exo‐Sap‐IT (Affymetrix‐USB Corporation) according to the manufacturer's instructions.

These PCR products were sequenced at Eurofins Genomics (Louisville, KY). Sequences for COI and cyt *b* were initially aligned with Sequencher (Gene Codes Corp.), with minor manual adjustments. Mitochondrial sequences were also translated to inspect them for ambiguous amino acids or stop codons; none were observed. The ITS1 sequences were aligned in the online version of MAFFT 7 (Katoh, Rozewicki, & Yamada, 2017) under the Q‐INS‐i algorithm with the default settings. Gaps were coded as characters following the simple method of Simmons and Ochoterena (2000) and appended to the nucleotide sequences using FastGap 1.2 (Borchsenius, 2009).

### 2.3 Genotyping by sequencing

To produce a much larger dataset from across the nuclear genome to refine our understanding of the identity of *A. arapahoe*, we used tunable genotyping by sequencing (GBS; Ott et al., 2017) on 180 specimens of capniid stoneflies (Data2Bio, Ames, Iowa). We focused on *A. arapahoe* and *A. decepta*, because of extensive haplotype overlap between these taxa, and *Capnia gracilaria* and *Capnura wanica*, because these were the most abundant confamilial stoneflies in this area (Fairchild et al., 2017) and the most likely contributors to heterospecific crosses. We also included representatives of all other members of the genus *Arsapnia* with unique haplotypes in the COI analysis and of most other capniid stoneflies present in Colorado (Supporting Information Table S1).

Initial sequencing produced ~501.7 M raw reads. After trimming reads with bases having PHRED quality scores of <15, ~440.6 M reads (mean length, 115 bp) remained. Consensus sequences were generated for *A. arapahoe* because of the lack of a suitable reference genome for alignment and SNP calling. Trimmed sequence reads from all samples were combined and normalized to a maximum of 50x coverage, using diginorm (Brown, Howe, Zhang, Pyrkosz, & Brom, 2012). The sequencing errors in the reads were then corrected using Fiona (Schulz et al., 2014). The coverage‐normalized and error‐corrected reads were condensed using CD‐HIT‐454 (Fu, Niu, Zhu, Wu, & Li, 2012) with ≥96% identity to form consensus clusters. Clusters with fewer than 10 component reads and shorter than 50 bp were discarded. Trimmed reads were aligned to the consensus reference sequence using GSNAP (Wu & Nacu, 2010). Confidently mapped reads were filtered if each mapped uniquely (≤2 mismatches every 36 bp and <5 bases for every 75 bp as tails) and were used for subsequent analyses.

Any site that was polymorphic (homozygous or heterozygous) relative to the reference genome sequence in at least one sample was considered a SNP. Putative homozygous and heterozygous SNPs were retained if the most common allele (or two alleles in heterozygotes) was supported by at least 80% of all the aligned reads covering that position, at least five unique reads supported the most common allele (or two most common alleles), and each polymorphic base had a PHRED base quality value ≥20. Polymorphisms in the first and last 3 bp of each read were ignored. Polymorphic sites were filtered further based on a minimum allele frequency of 1%, a constrained heterozygosity rate (ranging from zero to twice the product of the frequency of the two most common alleles), and a minimum call rate of 20%, that is, each locus was genotyped in 20% of all specimens.

### 2.4 Phylogenetic analyses

Our initial assessment of the validity and evolutionary position of *A. arapahoe* was based on four sets of analyses: (a) confinement to a statistical parsimony network independent of all other species and lack of haplotype sharing with other taxa; (b) reciprocal monophyly from all other stonefly species in the maximum‐likelihood phylogenetic trees of ITS1 and concatenated mtDNA sequences; (c) genetic differences commensurate with species‐level differences from all other taxa in the mitochondrial and ITS1 sequences; and (d) the presence of nucleotides diagnostic for this species in these sequences. First, we used TCS 1.21 (Clement, Posada, & Crandall, 2000) to construct 95% maximum parsimony networks based on COI sequences from field samples and public sequence libraries. Independent networks using this threshold have been regarded as representing single species, although this approach can underestimate species diversity because of the greater tendency to combine distinct taxa than to split a single taxon (Chen et al., 2010; Hart & Sunday, 2007). Sequences with ambiguous nucleotides were excluded from maximum parsimony networks to avoid spurious networks (Joly, Stevens, & van Vuuren, 2007). Second, we developed maximum‐likelihood phylogenetic trees for the ITS1 sequences and for concatenated sequences of COI and cyt *b*. Analyses were restricted to unique haplotypes which we identified using DAMBE version 6 (Xia, 2017). We used PartitionFinder 2.0 (Lanfear, Frandsen, Wright, Senfeld, & Calcott, 2016) to select the best‐fitting partitioning scheme as measured by AIC_c_, constrained to the suite of evolutionary models considered by RAxML (Stamatakis, 2014) and excluding all outgroups, that is, nonmembers of the *Arsapnia* group. There were six data subsets for the concatenated mitochondrial sequences based on codon position and gene, and two subsets for ITS1 based on nucleotides and recoded gap characters. Because RAxML will only consider a single evolutionary model for the entire suite of partitions, we compared AIC_c_ scores among maximum‐likelihood models using the GTR, GTRGAMMA, and GTRGAMMAI evolutionary models to choose a best model. We then ran RAxML version 8.1.21 implemented through RAxMLGUI (Silvestro & Michalak, 2012) and set for rapid bootstrapping (1,000 bootstraps) and a thorough ML search. Third, we calculated pairwise genetic distances between haplotypes of specimens in terminal clades and among species identified based on morphology using MEGA 7.0 (Kumar, Stecher, & Tamura, 2016) independently for the three gene regions. We based these calculations on uncorrected *p*‐distances because these have been shown to perform as well or better for detection of barcode gaps indicative of species‐level divergence than the more broadly used Kimura‐2‐parameter model (Collins, Boykin, Cruickshank, & Armstrong, 2012). We focused on distances within and between *A. arapahoe*, *A. decepta*, *Capnia gracilaria*, and *Capnura wanica*. For *A. decepta* and *Capnia gracilaria*, we considered samples from outside Colorado separately, to avoid inflating distance estimates by including cryptic, divergent lineages. Finally, we searched for pure diagnostic nucleotide characters (DeSalle, Egan, & Siddall, 2005) to identify *A. arapahoe*, that is, those nucleotide–position combinations found in all haplotypes of this species and in no other member of this family (Wong, Shivji, & Hanner, 2009).

### 2.5 SNP analyses

Results of these analyses were not consistent with the prevailing hypothesis for *A. arapahoe* as a distinct species despite pronounced morphological differences among males. Hence, we pursued GBS to evaluate an alternative hypothesis consistent with the analyses of the mitochondrial data (see below): *A. arapahoe* is the result of a heterospecific cross in which the female parent was *A. decepta*. Using the GBS data, we undertook three approaches to evaluate other taxa that might be involved and to determine whether hybridization was introgressive. First, using SNPs with a minimum call rate of 20% across the 180 specimens (*n* = 123,726 SNPs genotyped in 20% of all specimens and averaging >56 reads/SNP), we examined the number of shared SNPs between *A. arapahoe* and the three common syntopic species, with the prediction that potentially parental taxa would share the most SNPs with *A. arapahoe* (Huang & Knowles, 2014). Next, we used principle coordinate analysis in genalex 6 (Peakall & Smouse, 2006) and inferred potential parental taxa from their position relative to specimens of *A. arapahoe* in two‐dimensional coordinate space (Payseur & Rieseberg, 2016). We restricted these analyses to a single SNP at each locus with a minimum call rate of 90% (*n* = 1,788) to avoid issues with linkage and to minimize the influence of missing data on potential patterns in hybridization. For the likely parental taxa identified in these analyses, we examined SNPs present in every specimen (minimum call rate = 100%) that were fixed for alternate alleles in each taxon and thus potentially diagnostic to permit precise estimates of the levels of introgression and heterozygosity within individuals (Hohenlohe, Amish, Catchen, Allendorf, & Luikart, 2011). We examined the distribution of alleles at these loci only in male *A. arapahoe* because the two putative *A. arapahoe* females were assigned by mitochondrial sequences to members of other, morphologically similar species (Nelson & Baumann, 1989). Another female phenotypically identified as *A. decepta*, however, had a mitochondrial and nuclear genotype matching that of male *A. arapahoe* and was considered a female representative of this taxon (see below). This led to identification of sex chromosome‐linked SNPs, the allelic patterns of which differed from those in autosomal loci (Carmichael et al., 2012) but were consistent with the interpretation of the origin of *A. arapahoe*.

## 3 RESULTS

The 563‐nucleotide COI dataset consisted of 556 sequences constituting 135 haplotypes, the 918‐nucleotide cyt *b* dataset included 99 sequences representing 96 haplotypes, and the 428‐nucleotide ITS1 dataset contained 88 sequences and 67 haplotypes. The ITS1 sequences were bracketed by 27 nucleotides of r18S and 36 nucleotides of r5.8S; these regions were invariant in all taxa. In contrast, the ITS1 region was of variable length (273–308 nucleotides) because of the extensive insertions and deletions typical of noncoding regions.

**Figure 2:**
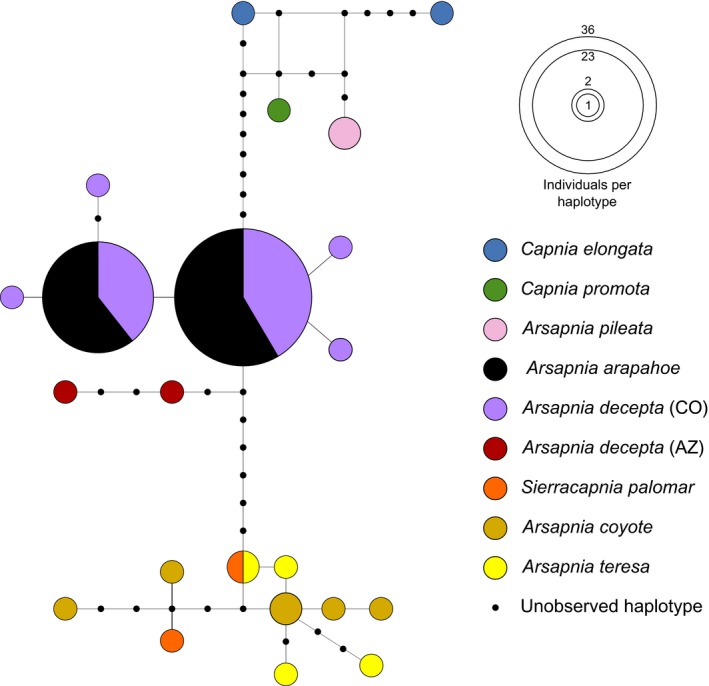
About 95% maximum parsimony network of cytochrome c oxidase subunit 1 sequences for haplotypes of capniid stoneflies from the clade representing the *Arsapnia* group. Only those specimens linked to the network containing *A. arapahoe* (*n* = 22) are shown. Each circle represents a haplotype, sizes are proportional to the number of individuals with that haplotype, and phenotypes associated with each haplotype are identified by color. Haplotype labels are in Supporting Information Tables S1 and S2. Each line segment represents a single mutation, and small black dots represent unobserved haplotypes. The remaining networks representing the *Arsapnia* group are in Supporting Information Figure S1

Analyses of 95% maximum parsimony networks of the *Arsapnia* group did not support recognizing *A. arapahoe* as a distinct taxon. Although 13 phenotypically identified species were included, the analysis produced only three separate networks (Figure 2, Supporting Information Figure S1). In the network with *A. arapahoe*, all specimens of that species shared haplotypes with *A. decepta*, and that network included four other species of *Arsapnia*, one species of *Sierracapnia*, and two species of *Capnia*. This network also included two specimens of *A. decepta* from Arizona, which were closely related to but distinct from those in Colorado. Interspecific patterns of relationships, however, were largely concordant with those in other sequence‐based analyses.

**Figure 3:**
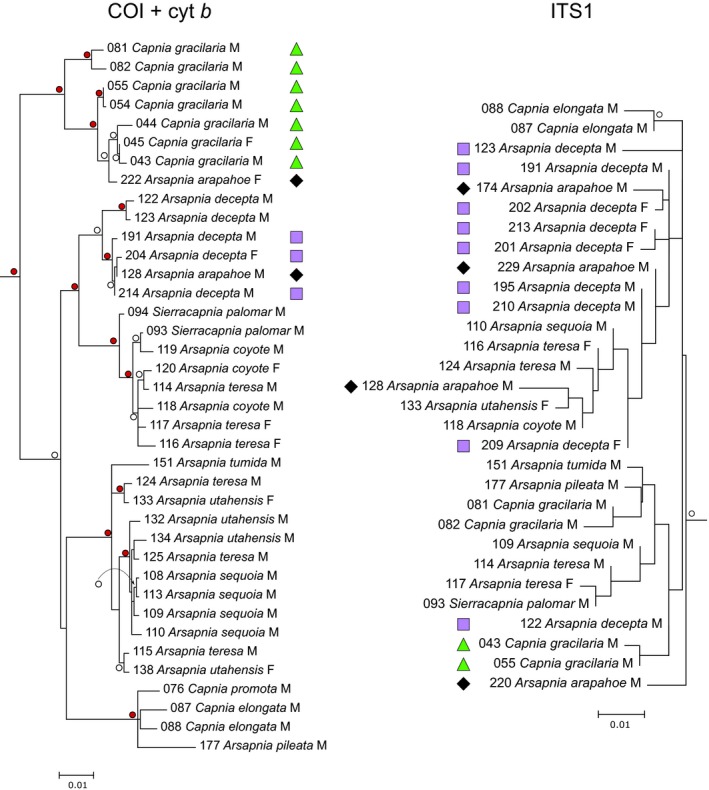
A portion of the best‐scoring phylogenetic tree inferred from a data‐partitioned maximum‐likelihood analysis (with 1,000 bootstrap replicates) of 91 sequences (1,419 nucleotides) of the concatenated mitochondrial genes cytochrome c oxidase subunit 1 and cytochrome b (COI + cyt *b*) and of 64 sequences (442 nucleotides and 87 gap‐coded positions) of the nuclear first internal transcribed spacer and adjacent portions of the r18S and r5.8S regions (ITS1). Only that portion of each tree representing the *Arsapnia* group is displayed. Sex of a specimen is indicated by M (male) or F (female). Symbols highlight specimens phenotypically identified as *A. arapahoe* (black diamonds), *A. decepta* (purple squares), or *Capnia gracilaria* (green triangles) in Colorado. Branches with bootstrap support >70% are labeled (○, bootstrap support 70%–90%; 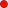, bootstrap support >90%). Branch lengths are proportional to the number of substitutions per site. For sequence numbers, see Supporting Information Table S1.

Both the mitochondrial and ITS1 phylogenies strongly supported the *Arsapnia* group (Figure 3). The mitochondrial phylogenies provided greater resolution at lower taxonomic levels, as would be expected because mitochondrial genes have smaller effective population sizes and thus diverge more rapidly relative to nuclear genes. In both analyses, *Capnia gracilaria* was supported as a member of (ITS1) or sister to (COI + cyt *b*) the remainder of the *Arsapnia* group. The terminal clades in the mitochondrial trees were not always concordant with the phenotypic identifications of specimens, especially of females (also see Supporting Information Table S1). This included two females phenotypically assigned to *A. arapahoe* that shared COI mitochondrial haplotypes with species in other genera: specimen 222 with *Capnia gracilaria* and specimen 225 with *Capnura wanica*. These sequences were unlikely to represent paralogous nuclear sequences because of their exact match to haplotypes of each of these taxa; thus, we considered them misidentified. Even ignoring these specimens, in neither phylogeny was *A. arapahoe* reciprocally monophyletic; it always (mitochondrial phylogeny) or usually (ITS1 phylogeny) occupied the same terminal clade as *A. decepta*.

Pairwise genetic distances supported a lack of divergence between *A. arapahoe* and *A. decepta*. Intraspecific differences in mitochondrial sequences between *A. arapahoe* and *A. decepta* from Colorado were minor (0.19%–0.31%), substantially smaller than those between *A. decepta* in Colorado and Arizona (1.11%–1.56%) or between *Capnia gracilaria* in Colorado and Oregon (1.74%–2.60%; Table 1). Mitochondrial differences between *A. arapahoe* and Colorado forms of *Capnia gracilaria* (2.96%–5.14%) and *Capnura wanica* (10.71%–13.79%) were also substantial. The ITS1 sequences revealed a slightly different pattern, in that the intraspecific variation in *A. arapahoe* (0.70%) was larger than its interspecific distance with respect to Colorado specimens of *A. decepta* (0.26%) and *Capnia gracilaria* (0.42%), yet still markedly smaller than the distance to *Capnura wanica* (7.18%).

**Table 1:** Mean pairwise genetic differences (%) among haplotypes within (on the diagonal) and between (below the diagonal) *Arsapnia arapahoe* and related or sympatric capniid stoneflies for two mitochondrial genes and one nuclear gene

Gene	Species	*Arsapnia arapahoe*	*Arsapnia decepta* (CO)	*Arsapnia decepta* (AZ)	*Capnia gracilaria* (CO)	*Capnia gracilaria* (OR)	*Capnura wanica*
COI	*Arsapnia arapahoe*	0.18					
	*Arsapnia decepta* (CO)	0.31	0.56				
	*Arsapnia decepta* (AZ)	0.80	1.11	0.53			
	*Capnia gracilaria* (CO)	2.96	3.18	3.13	0.73		
	*Capnia gracilaria* (OR)	2.84	3.06	3.02	1.74	0.18	
	*Capnura wanica*	10.71	10.80	10.87	10.77	10.53	0.36
cyt *b*	*Arsapnia arapahoe*	0					
	*Arsapnia decepta* (CO)	0.19	0.23				
	*Arsapnia decepta* (AZ)	1.52	1.56	0.23			
	*Capnia gracilaria* (CO)	5.14	5.18	5.43	0.70		
	*Capnia gracilaria* (OR)	4.67	4.71	5.14	2.60	1.05	
	*Capnura wanica*	13.79	13.67	14.14	15.60	15.25	1.17
ITS1	*Arsapnia arapahoe*	0.70					
	*Arsapnia decepta* (CO)	0.26	0.09				
	*Arsapnia decepta* (AZ)	0.53	0.40	0.53			
	*Capnia gracilaria* (CO)	0.42	0.31	0.53	0		
	*Capnia gracilaria* (OR)	0.79	0.70	0.79	0.79	0	
	*Capnura wanica*	7.18	7.12	6.73	6.86	6.86	0

There were no single nucleotides in the COI or ITS1 analyses that were diagnostic for *A. arapahoe* or for the combination of *A. arapahoe* and *A. decepta*. A single nucleotide in the cyt *b* sequences was diagnostic for this combination (position 213, third codon, C; all other taxa, A or T). The public cyt *b* database, however, was represented by relatively few sequences and taxa, and this diagnostic position may have been represented in other taxa had larger numbers been available to examine.

**Figure 4:**
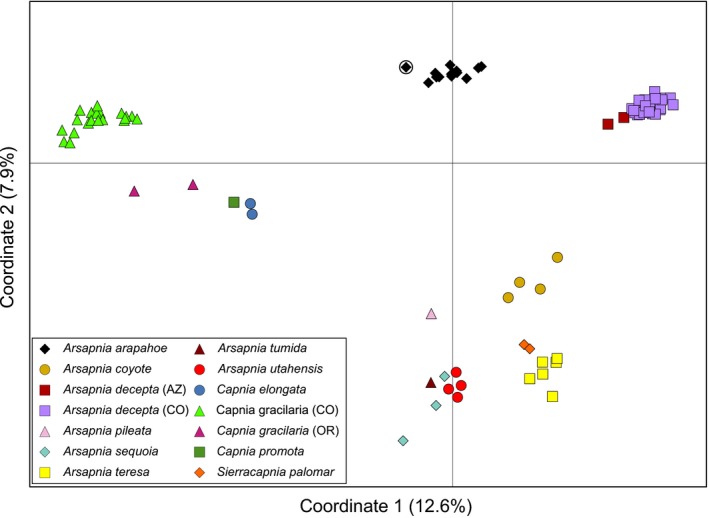
Scatterplot of specimens of the *Arsapnia* group on the first two principle coordinates derived from genetic distances based on 1,788 SNPs from 98 individuals. Samples are coded by phenotypically identified species, except a female (specimen 279) phenotypically identified as *A. decepta* with a mitochondrial and SNP genotype of *A. arapahoe* (circled)

Analyses of genome‐wide SNPs clarified the origin of *A. arapahoe*. Of the 123,726 SNPs across the entire sample of specimens with a minimum call rate of 20%, 54,293 were present in *A. arapahoe*. Large numbers of these were shared by *A. decepta* (20,965 SNPs) and *C. gracilaria* (18,925 SNPs), but not by *Capnura wanica* (62 SNPs), indicating that the latter species did not contribute to the *A. arapahoe* lineage. The principle coordinate analyses based on SNPs with a 90% minimum call rate positioned *A. arapahoe* midway between *A. decepta* and *Capnia gracilaria*, with all three taxa distantly related to most other members of the *Arsapnia* group (Figure 4). Specimen 279, originally identified as a female *A. decepta*, grouped with *A. arapahoe* and shared a mitochondrial haplotype with other *A. arapahoe* (Supporting Information Table S1). Thus, we considered this specimen to be a female *A. arapahoe*.

**Figure 5:**
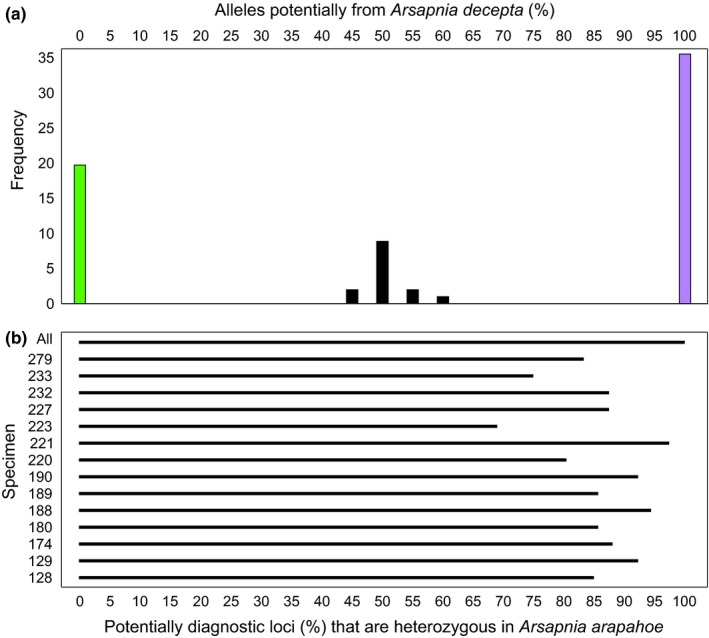
Patterns of genome‐wide SNPs (*n* = 94) in *Arsapnia arapahoe* (*n* = 14; black bars) that were fixed for alternate (and potentially diagnostic) alleles in *Capnia gracilaria* (*n* = 20; green bars) and *A. decepta* (*n* = 36; purple bars) in samples of these species from Colorado. (a) The percentage of alleles potentially diagnostic for *A. decepta*. That value subtracted from 100 equals the percentage of loci potentially derived from *Capnia gracilaria*. The central peak represents *A. arapahoe* with equal contributions of alleles from both potential parental species. (b) The percentage of loci at which *A. arapahoe* specimens are heterozygous. For 60 presumably autosomal loci, all *A. arapahoe* specimens (all) were heterozygous at all loci, indicating that alleles at these loci are diagnostic. For an additional 34 nondiagnostic autosomal SNPs, percentages of loci that were heterozygous are given for each specimen. Not shown are results for 51 sex‐linked SNPs for which all male *A. arapahoe* (specimens 128–233) are fixed for the alleles from *A. decepta*. The single female *A. arapahoe* (specimen 279) was heterozygous for each of these loci

Candidate diagnostic SNPs demonstrated that *A. arapahoe* was of hybrid origin. When considering all 145 SNPs fixed for different alleles in *A. decepta* and *Capnia gracilaria*, 60 were diagnostic when using *A. arapahoe* as a reference, that is, every specimen of *A. arapahoe* had half of its alleles from each of these species, and each SNP position was heterozygous (Figure 5). For an additional 51 SNPs, every male *A. arapahoe* was homozygous for the alleles from *A. decepta*, whereas the single female of this species was heterozygous for alleles from both parental species, indicating that these were sex‐linked SNPs and consistent with a form of X0 sex determination (Blackmon, Ross, & Bachtrog, 2016). The remaining SNPs were nondiagnostic or may have exhibited occasional genotyping errors, yet their allelic distributions were demonstrative of a first‐generation hybrid origin of *A. arapahoe*. For the nondiagnostic SNPs, about half of the alleles in *A. arapahoe* were from *A. decepta* (mean 53.6%, range, 48.5%–59.4%) and specimens were heterozygous at the majority of these SNPs (mean 85.8%, range 73.5%–96.8%). No individual of *A. arapahoe* for which we had SNP data had a genotype indicative of any level of introgression resulting from backcrosses to either parental species (in which case an individual would have ≥75% of the diagnostic alleles of one parent) or mating between hybrid individuals (in which case levels of heterozygosity would be ≤25%; Figure 5). Finally, all SNPs fixed for single allele in *A. arapahoe* (*n* = 1,275) were shared with *A. decepta* and *Capnia gracilaria*, that is, no SNPs were diagnostic for *A. arapahoe*.

## 4 DISCUSSION

Collectively, the genetic analyses of specimens from nine separate locations demonstrated that *A. arapahoe* consisted of individuals that were the first‐generation progeny of female *A. decepta* and male *Capnia gracilaria*. The phylogenetic analyses of two mitochondrial genes and one nuclear gene did not delineate *A. arapahoe* as a taxon distinct from its more common, widespread, and sympatric congener, *A. decepta*. Specimens of *A. arapahoe* often shared haplotypes with *A. decepta* and always occupied the same terminal clades. Interspecific differences between *A. arapahoe* and *A. decepta* were typical of differences found within, not between, other species of stoneflies, and were comparable to intraspecific mitochondrial variation throughout much of the animal kingdom (Goldstein & DeSalle, 2010), providing no evidence of any degree of lineage sorting. Although analyses of sequences of ITS1 were less informative, they did reveal monophyly among the *Arsapnia* group, which included *Capnia gracilaria*. In contrast, analyses of genome‐wide, autosomal and sex‐linked SNPs identified ongoing hybridization as the source of *A. arapahoe*, delineated the two parental taxa and (in conjunction with the mitochondrial data) the directionality of matings, and revealed that hybridization was nonintrogressive. This understanding offers a cautionary tale for practitioners of taxonomy and conservation of this and similar groups of little‐studied organisms.

We note that neither DNA barcoding nor morphological diagnosis of *Arsapnia arapahoe* failed wholly; rather, neither could fully elucidate the process that generated this species hypothesis. The mitochondrial contribution of female *A. decepta* to *A. arapahoe* was indicated by the absence of divergence between them, but was moot on whether this was from hybridization, recent divergence, or a faulty taxonomy. In retrospect, the combination in male *A. arapahoe* of many *Arsapnia*‐like anatomical characters (Murányi et al., 2014) and a relatively long epiproct reminiscent of *Capnia gracilaria* (and other species of *Capnia*; Nelson & Baumann, 1989) might have been interpreted as evidence of the hybrid origin of *A. arapahoe*, albeit one difficult to recognize amidst a background of morphological variability (Baumann & Stark, 2017). Regardless, the rise of genetic and genomic tools has made the detection of hybridization relatively straightforward, which in turn has revealed that hybridization and even hybrid speciation are a regular occurrence among many insect taxa (The Heliconius Research Consortium, 2012). Ours and other recent studies (Boumans & Tierno de Figueroa, 2016; Dussex, Chuah, & Waters, 2016) extend observations of these phenomena to Plecoptera across the globe and require their consideration in taxonomic and ecological studies, especially in instances of morphological and molecular (or cytonuclear) disagreement. Although we focused on the origins of *A. arapahoe*, other taxa included in our analyses have patterns that strongly parallel those that were indicative of hybridization (Figures 2 and 3). For example, male *Sierracapnia palomar* have a long, narrow epiproct markedly different from other members of *Sierracapnia* (Bottorff & Baumann, 2015), the species is a local endemic that is geographically disjunct from other congeners, and its haplotypes cluster with species of *Arsapnia*. Evaluating whether hybridization influences this species hypothesis, however, will require focused sampling of additional capniid stoneflies in and around its small range in central California.

Zoogeographic patterns suggest that the hybrids constituting *A. arapahoe* may be relatively restricted in their distribution. *Arsapnia decepta* is primarily a species of small and sometimes intermittent streams of the southwestern United States and adjacent Mexico that makes its most northerly advance along the Colorado Front Range. In contrast, *Capnia gracilaria* appears to occur in larger streams and the majority of its range is from South Dakota to Oregon and north through the Rocky Mountains to Alaska, with scattered observations in Arizona, New Mexico, and the Great Basin (Baumann, Sheldon, & Bottorff, 2017; DeWalt, Maehr, Neu‐Becker, & Stueber, 2018; Kondratieff & Baumann, 2002; Nelson & Baumann, 1989). This limits potential sympatry to basins in the central and southern U.S. Rocky Mountains, and even their areas of contact may be limited. The hybrid zones in northern Colorado streams are highly local (Fairchild et al., 2017), hinting at strong abiotic controls on the distribution of each species that restrict hybridization to short reaches of small streams where adult emergence of each species is synchronized by elevation or stream temperature. If so, it should be possible to predict the locations of hybrid zones in areas that have not yet been sampled or to forecast environmentally driven hybrid zone dynamics (cf. Young et al., 2016).

Confirmation of the presence of these hybrid zones will rely almost entirely on finding rare male *A. arapahoe*. In sampling targeted at *A. arapahoe*, only 41 of 26,170 specimens were morphologically identified as that taxon, and all were male (Fairchild et al., 2017). Females may have been equally represented, but the lack of distinguishing characteristics among many female capniid stoneflies may result in fewer being identified or being identified correctly. Genotyping of putative female *A. arapahoe* in the present study (and previously; Heinold et al., 2014) revealed that they possessed mitochondrial haplotypes identical to those of other capniid stoneflies. Similarly, a female identified as *A. decepta* based on morphological characters represented the only molecularly supported example of a female *A. arapahoe*. A third misidentification involved one of the 14 females phenotyped as *A. decepta*, which phylogenetically clustered with *Capnia coloradensis* Claassen. This particular error has precedent; Nelson and Baumann (1989) remarked that two observations of female *A. decepta* in locations geographically disjunct from their core range were likely attributable to misidentifications of *Capnia coloradensis*. Even for taxonomic experts, reliable identification of female capniid stoneflies may not be possible without resorting to molecular methods. This suggests some caution in accepting identifications of morphologically ambiguous individuals in museum collections unless supported by genetic data, and in assuming that reference sequences in public databases are correctly identified (Kvist, Oceguera‐Figueroa, Siddall, & Erséus, 2010), especially if the sex of those specimens is not recorded.

Nevertheless, such collections are the foundation of ecological, genetic, and taxonomic exploration for many taxa. Our interrogation of the status of *A. arapahoe* was motivated by the conflict between morphological and molecular interpretations of this species and made possible by a comprehensive museum collection. Despite recent calls to bolster the ranks of traditional taxonomists and the collections, they steward (Morrison, Sillett, Funk, Ghalambor, & Rick, 2017), the taxonomic impediment remains. There are too few taxonomists, and they are confronted by waves of genetic data simultaneously suggesting candidate species and challenging long‐standing species hypotheses. Technological advances that facilitate recovering genome‐wide data for many species, including from environmental samples for which detected taxa are never observed (Deiner et al., 2017), and the increasingly sophisticated algorithms for genetically driven species delimitation (Luo, Ling, Ho, & Zhu, 2018), make it likely that species discovery and revision will increasingly be crowd‐sourced to nontaxonomists such as ecologists and geneticists. The lack of consensus among species concepts and the variation in how genetic data are interpreted (Sukumaran & Knowles, 2017), however, suggest that an integrative taxonomy using multiple data sources (Padial, Miralles, De la Riva, & Vences, 2010) should be the standard, and emphasizes that expertise in morphological assessment remains indispensable (Zhou et al., 2016). Robust taxonomies also rely on the taxonomist's exploration of novel habitats and the ecologist's systematic inventory of representative ones (Sheldon, 2016) to produce the comprehensive zoogeographical sampling that is paramount to delimiting and describing biodiversity (Young, McKelvey, Pilgrim, & Schwartz, 2013).

Such an intensive approach may only be practical for taxa, or the hypotheses they represent, that are of intense conservation interest. This level of attention is being directed at many groups of freshwater species, including stoneflies, because they are disproportionately represented in lists of imperiled taxa (Strayer & Dudgeon, 2010; Williams et al., 2011). In the U.S., federal listing under the Endangered Species Act is typically reserved for those rare and declining taxa, or the distinct populations segments thereof, that appear most at risk. The highly restricted range and limited abundance of *A. arapahoe*, in light of the existing and proposed developments in this region, met those requirements and elevated this species to candidacy for listing (U.S. Fish and Wildlife Service, 2012). This status, however, rests on the notion that it represents a valid taxon to which a name may be applied. With respect to animal taxa of hybrid origin, the International Commission on Zoological Nomenclature has concluded that the zoological code does not provide for the naming of hybrids (http://iczn.org/content/article-301), except perhaps in instances of hybrid speciation leading to self‐sustaining lineages. We have no evidence, however, that *A. arapahoe* constitutes such a lineage; instead, the lack of later‐generation hybrids or backcrosses is indicative of nonintrogressive hybridization. Consequently, the individuals formerly recognized at *A. arapahoe* instead should be referred to as first‐generation hybrids between female *A. decepta* and male *Capnia gracilaria* (Frank‐Thorston Krell, International Commission on Zoological Nomenclature, personal communication). More broadly, these individuals are further evidence of the ubiquity of hybridization even between species thought to be reproductively isolated by morphology and behavior, and a reminder to consider this phenomenon as a potential source of variation in taxonomic and phylogenetic studies. We have little doubt that further instances of unrecognized hybridization, and its taxonomic consequences, will become apparent as genomic exploration of understudied groups continues.

## CONFLICT OF INTEREST

None declared.

## AUTHOR CONTRIBUTIONS

Designed research: MKY, KLP, MF, MKS; Performed research: MKY, RJS, KLP, MF; Analyzed data: MKY; Wrote the paper: MKY, RJS, KLP, MF, MKS.

## Data Availability

Sequences of COI, cyt *b*, and ITS1 were deposited as GenBank accessions MK275688–276068. The ITS1 alignment is available in Dryad (https://doi.org/10.5061/dryad.c317v31).
