# Supplementary material for: Integrative taxonomy refutes a species hypothesis: The asymmetric hybrid origin of Arsapnia arapahoe (Plecoptera, Capniidae)
Source: Ecol Evol. 2019 Jan 13;9(3):1364–77. doi: 10.1002/ece3.4852 (PMC6374720; doi:10.1002/ece3.4852)

Table S1. Specimens sequenced at COI, cyt *b*, or ITS1, or genotyped by sequencing. All specimens were courtesy of the C.P. Gillette Museum of Arthropod Diversity, Colorado State University, Fort Collins, Colorado.

|  |  |  | Genetic data^3^ | | | |  |  |  |  |  |
| --- | --- | --- | --- | --- | --- | --- | --- | --- | --- | --- | --- |
| Taxon | Code^1^ | Sex^2^ | COI | cyt *b* | ITS1 | GBS | Site | State | Latitude | Longitude | Date |
| *Capnia confusa* | 1 | M | X | X | X | X | Elkhorn Cr | CO | 40.6978 | 105.4408 | 4/25/2013 |
| *Capnia confusa* | 2 | M | X | X | X | X | Elkhorn Cr | CO | 40.6978 | 105.4408 | 4/25/2013 |
| *Capnia confusa* | 3 | F | X | X | X | X | Elkhorn Cr | CO | 40.6978 | 105.4408 | 4/25/2013 |
| *Capnia confusa* | 4 | F | X | X | X | X | Elkhorn Cr | CO | 40.6978 | 105.4408 | 4/25/2013 |
| *Capnia vernalis* | 5 | M | X | X | X | X | Williams Fork | CO | 40.3728 | 107.6146 | 4/11/2015 |
| *Capnia vernalis* | 6 | F | X | X | X | X | Williams Fork | CO | 40.3728 | 107.6146 | 4/11/2015 |
| *Capnia vernalis* | 7 | F | X |  |  |  | Williams Fork | CO | 40.3728 | 107.6146 | 4/11/2015 |
| *Utacapnia logana* | 8 | M | X | X | X | X | Kennedy Gul | CO | 39.4665 | 105.2395 | 2/20/2014 |
| *Utacapnia logana* | 9 | M | X | X | X | X | Kennedy Gul | CO | 39.4665 | 105.2395 | 2/20/2014 |
| *Utacapnia logana* | 10 | F | X |  |  |  | Kennedy Gul | CO | 39.4665 | 105.2395 | 2/20/2014 |
| *Utacapnia logana* | 11 | F | X | X | X | X | Kennedy Gul | CO | 39.4665 | 105.2395 | 2/20/2014 |
| *Isocapnia vedderensis* | 12 | F | X | X | nd | X | Cedar Cr | CO | 40.4661 | 105.2903 | 4/21/2014 |
| *Paracapnia angulata* | 13 | M | X | X | nd | X | Elkhorn Cr | CO | 40.6978 | 105.4408 | 4/20/2013 |
| *Paracapnia angulata* | 14 | M | X |  |  |  | Elkhorn Cr | CO | 40.6978 | 105.4408 | 4/20/2013 |
| *Paracapnia angulata* | 15 | F | X |  |  |  | Elkhorn Cr | CO | 40.6978 | 105.4408 | 4/20/2013 |
| *Paracapnia angulata* | 16 | F | X |  |  |  | Elkhorn Cr | CO | 40.6978 | 105.4408 | 4/20/2013 |
| *Capnura wanica* | 17 | M | X | X | X | X | Sheep Cr | CO | 40.5706 | 105.3483 | 3/14/2013 |
| *Capnura wanica* | 18 | M | X | X | X | X | Sheep Cr | CO | 40.5706 | 105.3483 | 3/14/2013 |
| *Capnura wanica* | 19 | F | X |  |  | X | Sheep Cr | CO | 40.5706 | 105.3483 | 3/14/2013 |
| *Capnura wanica* | 20 | F | X |  |  | X | Sheep Cr | CO | 40.5706 | 105.3483 | 3/14/2013 |
| *Capnura fibula* | 21 | M | X | X | X | X | Purgatoire R | CO | 37.1689 | 104.5097 | 2/18/2015 |
| *Capnura fibula* | 22 | M | X | nd | X | X | Purgatoire R | CO | 37.1689 | 104.5097 | 2/18/2015 |
| *Capnura fibula* | 23 | F | X |  |  |  | Purgatoire R | CO | 37.1689 | 104.5097 | 2/18/2015 |
| *Capnura fibula* | 24 | F | X | nd | X | X | Purgatoire R | CO | 37.1689 | 104.5097 | 2/18/2015 |
| *Mesocapnia frisoni* | 29 | M | X | X | X | X | Little Thompson R | CO | 40.2576 | 105.1592 | 3/17/2013 |
| *Mesocapnia frisoni* | 30 | M | X | X | nd | X | Little Thompson R | CO | 40.2576 | 105.1592 | 3/17/2013 |
| *Paracapnia angulata* | 31 | M | X |  |  |  | Dale Cr | CO | 40.8967 | 105.3728 | 3/31/2013 |
| *Paracapnia angulata* | 32 | M | X |  |  |  | Dale Cr | CO | 40.8967 | 105.3728 | 3/31/2013 |
| *Paracapnia angulata* | 33 | F | X |  |  |  | Dale Cr | CO | 40.8967 | 105.3728 | 3/31/2013 |
| *Paracapnia angulata* | 34 | F | X |  |  |  | Dale Cr | CO | 40.8967 | 105.3728 | 3/31/2013 |
| *Capnia coloradensis* | 35 | M | X | X | X |  | Tenmile Cr | CO | 39.5746 | 106.1107 | 3/17/2014 |
| *Capnia coloradensis* | 36 | M | X |  |  |  | Tenmile Cr | CO | 39.5746 | 106.1107 | 3/17/2014 |
| *Capnia coloradensis* | 38 | F | X |  |  | X | Tenmile Cr | CO | 39.5746 | 106.1107 | 3/17/2014 |
| *Capnura wanica* | 39 | M | X |  |  | X | Elkhorn Cr | CO | 40.6978 | 105.4408 | 3/17/2013 |
| *Capnura wanica* | 40 | M | X |  |  | X | Elkhorn Cr | CO | 40.6978 | 105.4408 | 3/17/2013 |
| *Capnura wanica* | 41 | F | X | X | X | X | Elkhorn Cr | CO | 40.6978 | 105.4408 | 3/17/2013 |
| *Capnura wanica* | 42 | F | X |  |  |  | Elkhorn Cr | CO | 40.6978 | 105.4408 | 3/17/2013 |
| *Capnia gracilaria* | 43 | M | X | X | X | X | Elkhorn Cr | CO | 40.6978 | 105.4408 | 4/20/2013 |
| *Capnia gracilaria* | 44 | M | X | X | X | X | Elkhorn Cr | CO | 40.6978 | 105.4408 | 4/20/2013 |
| *Capnia gracilaria* | 45 | F | X | X | X | X | Elkhorn Cr | CO | 40.6978 | 105.4408 | 4/20/2013 |
| *Capnia gracilaria* | 46 | F | X |  |  |  | Elkhorn Cr | CO | 40.6978 | 105.4408 | 4/20/2013 |
| *Eucapnopsis brevicauda* | 47 | M | X | X | nd | X | S Saint Vrain Cr | CO | 40.1246 | 105.4423 | 6/1/2013 |
| *Eucapnopsis brevicauda* | 48 | M | X | X | X | X | S Saint Vrain Cr | CO | 40.1246 | 105.4423 | 6/1/2013 |
| *Isocapnia vedderensis* | 51 | M | X | X | X | X | Cedar Cr | CO | 40.4661 | 105.2903 | 4/21/2014 |
| *Utacapnia logana* | 52 | M | X |  |  |  | Elkhorn Cr | CO | 40.6978 | 105.4408 | 3/20/2013 |
| *Utacapnia logana* | 53 | M | X | X | X | X | Elkhorn Cr | CO | 40.6978 | 105.4408 | 3/20/2013 |
| *Capnia gracilaria* | 54 | M | X | X | nd | X | Bummers Gul | CO | 40.0183 | 105.3675 | 2/28/2014 |
| *Capnia gracilaria* | 55 | M | X | X | X | X | Bummers Gul | CO | 40.0183 | 105.3675 | 2/28/2014 |
| *Capnia nana* | 56 | M | X | X | X | X | Pitcher Cr | MT | 45.6552 | 110.9416 | 2/15/2014 |
| *Capnia nana* | 57 | M | X | X | X | X | Pitcher Cr | MT | 45.6552 | 110.9416 | 2/15/2014 |
| *Capnia coloradensis* | 58 | M | X | X | X |  | Trib to Granite Cr | AK | 60.7882 | 149.2115 | 4/24/2016 |
| *Capnia coloradensis* | 59 | M | X |  |  | X | Trib to Granite Cr | AK | 60.7882 | 149.2115 | 4/24/2016 |
| *Isocapnia integra* | 60 | M | X | X | nd | X | Moose Cr | AK | 61.6828 | 149.0471 | 4/27/2016 |
| *Capnia confusa* | 61 | M | X | X | X | X | EF Sixmile Cr | AK | 60.7317 | 149.3400 | 4/24/2016 |
| *Capnia confusa* | 62 | F | X |  |  |  | EF Sixmile Cr | AK | 60.7317 | 149.3400 | 4/24/2016 |
| *Capnia gracilaria* | 63 | M | X |  |  |  | NF Campbell Cr | AK | 61.1666 | 149.6876 | 4/26/2016 |
| *Capnia gracilaria* | 64 | M | X |  |  |  | NF Campbell Cr | AK | 61.1666 | 149.6876 | 4/26/2016 |
| *Capnia melia* | 65 | M | X | X | X | X | Wyatt Cr | OR | 43.6391 | 122.6200 | 3/15/2016 |
| *Capnia licina* | 66 | M | X | X | X | X | Salmon R | OR | 45.2609 | 121.7137 | 3/12/2016 |
| *Eucapnopsis brevicauda* | 67 | M | X | X | nd |  | Mill Cr | OR | 44.4396 | 120.5806 | 3/14/2016 |
| *Eucapnopsis brevicauda* | 68 | M | X |  |  |  | Mill Cr | OR | 44.4396 | 120.5806 | 3/14/2016 |
| *Eucapnopsis brevicauda* | 69 | F | X | X | nd | X | Mill Cr | OR | 44.4396 | 120.5806 | 3/14/2016 |
| *Eucapnopsis brevicauda* | 70 | F | X |  |  | X | Mill Cr | OR | 44.4396 | 120.5806 | 3/14/2016 |
| *Isocapnia spenceri* | 71 | M | X | nd | nd | X | McKenzie R | OR | 44.1113 | 123.0476 | 3/15/2016 |
| *Isocapnia spenceri* | 72 | M | X | nd | nd | X | McKenzie R | OR | 44.1113 | 123.0476 | 3/15/2016 |
| *Taeniopteryx nivalis* | 73 | M | X | X | nd | X | Grande Ronde R | OR | 45.3419 | 118.2364 | 3/13/2016 |
| *Taeniopteryx nivalis* | 74 | M | X | X | nd | X | Grande Ronde R | OR | 45.3419 | 118.2364 | 3/13/2016 |
| *Capnia promota* | 75 | M | H20 |  |  |  | McKenzie R | OR | 44.1113 | 123.0476 | 3/15/2016 |
| *Capnia promota* | 76 | M | X | X | nd | X | McKenzie R | OR | 44.1113 | 123.0476 | 3/15/2016 |
| *Eucapnopsis brevicauda* | 77 | M | X |  |  |  | Brice Cr | OR | 43.6747 | 122.7296 | 3/15/2016 |
| *Eucapnopsis brevicauda* | 78 | M | X | X | nd | X | Brice Cr | OR | 43.6747 | 122.7296 | 3/15/2016 |
| *Eucapnopsis brevicauda* | 80 | F | X | X | nd | X | Brice Cr | OR | 43.6747 | 122.7296 | 3/15/2016 |
| *Capnia gracilaria* | 81 | M | X | X | X | X | Lemon Gul | OR | 44.4195 | 120.6262 | 3/14/2016 |
| *Capnia gracilaria* | 82 | M | X | X | X | X | Lemon Gul | OR | 44.4195 | 120.6262 | 3/14/2016 |
| *Capnia glabra* | 83 | M | X | X | X |  | Frazier Cr | OR | 45.1630 | 118.6444 | 3/13/2016 |
| *Capnia glabra* | 84 | M | X |  |  | X | Frazier Cr | OR | 45.1630 | 118.6444 | 3/13/2016 |
| *Capnia californica* | 85 | M | X | X | X | X | Trib to Redwood Cr | CA | 40.8742 | 123.7558 | 3/3/2016 |
| *Capnia californica* | 86 | M | X |  |  |  | Trib to Redwood Cr | CA | 40.8742 | 123.7558 | 3/3/2016 |
| *Capnia elongata* | 87 | M | H22 | X | X | X | Clackamas R | OR | 45.1956 | 122.2169 | 3/16/2016 |
| *Capnia elongata* | 88 | M | H19 | X | X | X | Clackamas R | OR | 45.1956 | 122.2169 | 3/16/2016 |
| *Paracapnia disala* | 89 | M | X | X | nd | X | Trib to Parker Cr | OR | 44.4975 | 123.5565 | 3/16/2016 |
| *Paracapnia disala* | 90 | F | X |  |  |  | Trib to Parker Cr | OR | 44.4975 | 123.5565 | 3/16/2016 |
| *Capnura anas* | 91 | M | X | X | nd | X | Tybow Canyon Cr | OR | 45.2318 | 118.4617 | 3/13/2016 |
| *Capnura anas* | 92 | M | X | X | X | X | Tybow Canyon Cr | OR | 45.2318 | 118.4617 | 3/13/2016 |
| *Sierracapnia palomar* | 93 | M | H16 | X | X | X | Fry Cr | CA | 33.3440 | 116.8800 | 1/11/2012 |
| *Sierracapnia palomar* | 94 | M | H09 | X | nd | X | Fry Cr | CA | 33.3440 | 116.8800 | 1/11/2012 |
| *Capnia petila* | 95 | M | X | X | X | X | Daly Cr | MT | 46.1871 | 113.8922 | 3/1/2016 |
| *Capnia petila* | 96 | F | X | X | X | X | Daly Cr | MT | 46.1871 | 113.8922 | 3/1/2016 |
| *Bolshecapnia missiona* | 97 | M | X | X | nd | X | Daly Cr | MT | 46.1871 | 113.8922 | 3/1/2016 |
| *Bolshecapnia missiona* | 98 | M | X | X | nd |  | Daly Cr | MT | 46.1871 | 113.8922 | 3/1/2016 |
| *Bolshecapnia missiona* | 99 | F | X |  |  | X | Daly Cr | MT | 46.1871 | 113.8922 | 3/1/2016 |
| *Bolshecapnia missiona* | 100 | F | X | X | X | X | Daly Cr | MT | 46.1871 | 113.8922 | 3/1/2016 |
| *Capnia confusa* | 101 | M | X | nd | X | X | Grande Ronde R | OR | 45.3419 | 118.2364 | 3/13/2016 |
| *Capnia confusa* | 102 | M | X | X | X | X | Grande Ronde R | OR | 45.3419 | 118.2364 | 3/13/2016 |
| *Capnia excavata* | 103 | M | X | X | X | X | Brice Cr | OR | 43.6747 | 122.7296 | 3/15/2016 |
| *Isocapnia spenceri* | 105 | M | X |  |  |  | Willamette R | OR | 44.5387 | 123.2474 | 3/16/2016 |
| *Isocapnia grandis* | 106 | M | X |  |  |  | Willamette R | OR | 44.5387 | 123.2474 | 3/16/2016 |
| *Isocapnia grandis* | 107 | F | X | X | X | X | Willamette R | OR | 44.5387 | 123.2474 | 3/16/2016 |
| *Arsapnia sequoia* | 108 | M | X | X | X | X | Ackerson Cr | CA | 37.8326 | 119.8501 | 2/7/2016 |
| *Arsapnia sequoia* | 109 | M | X | X | X | X | Ackerson Cr | CA | 37.8326 | 119.8501 | 2/7/2016 |
| *Arsapnia sequoia* | 110 | M | X | X | X | X | Rush Cr | CA | 37.8090 | 119.8849 | 2/7/2016 |
| *Arsapnia sequoia* | 111 | M | X |  |  |  | Rush Cr | CA | 37.8090 | 119.8849 | 2/7/2016 |
| *Arsapnia sequoia* | 112 | M | X |  |  |  | Soldier Cr | CA | 37.8062 | 119.9195 | 2/7/2016 |
| *Arsapnia sequoia* | 113 | M | X | X | X | X | Soldier Cr | CA | 37.8062 | 119.9195 | 2/7/2016 |
| *Arsapnia teresa* | 114 | M | H10 | X | X | X | Ice House Cr | CA | 34.2505 | 117.6362 | 2/5/2016 |
| *Arsapnia teresa* | 115 | M | X | X | X | X | Ice House Cr | CA | 34.2505 | 117.6362 | 2/5/2016 |
| *Arsapnia teresa* | 116 | F | H15 | X | X | X | Ice House Cr | CA | 34.2505 | 117.6362 | 2/5/2016 |
| *Arsapnia teresa* | 117 | F | H14 | X | X | X | Ice House Cr | CA | 34.2505 | 117.6362 | 2/5/2016 |
| *Arsapnia coyote* | 118 | M | H12 | X | X | X | Seeley Cr | CA | 34.2719 | 117.3046 | 2/5/2016 |
| *Arsapnia coyote* | 119 | M | H17 | X | X | X | Seeley Cr | CA | 34.2719 | 117.3046 | 2/5/2016 |
| *Arsapnia coyote* | 120 | F | H11 | X | X | X | Seeley Cr | CA | 34.2719 | 117.3046 | 2/5/2016 |
| *Arsapnia coyote* | 121 | F | H11 |  |  |  | Seeley Cr | CA | 34.2719 | 117.3046 | 2/5/2016 |
| *Arsapnia decepta* | 122 | M | H08 | X | X | X | Christopher Cr | AZ | 34.1877 | 111.0230 | 2/3/2016 |
| *Arsapnia decepta* | 123 | M | H07 | X | X | X | Christopher Cr | AZ | 34.1877 | 111.0230 | 2/3/2016 |
| *Arsapnia teresa* | 124 | M | X | X | X | X | San Antonio Cr | CA | 34.2553 | 117.6480 | 2/5/2016 |
| *Arsapnia teresa* | 125 | M | X | X | nd | X | San Antonio Cr | CA | 34.2553 | 117.6480 | 2/5/2016 |
| *Arsapnia teresa* | 126 | F | X |  |  |  | San Antonio Cr | CA | 34.2553 | 117.6480 | 2/5/2016 |
| *Arsapnia teresa* | 127 | F | H09 |  |  |  | San Antonio Cr | CA | 34.2553 | 117.6480 | 2/5/2016 |
| *Arsapnia arapahoe* | 128 | M | H04 | X | X | X | Young Gul | CO | 40.6886 | 105.3473 | 2/12/2016 |
| *Arsapnia arapahoe* | 129 | M | H01 |  |  | X | Young Gul | CO | 40.6886 | 105.3473 | 2/12/2016 |
| *Arsapnia sequoia* | 130 | M | X |  |  |  | SF Tuolumne R | CA | 37.8113 | 119.9367 | 2/7/2016 |
| *Arsapnia sequoia* | 131 | M | X |  |  |  | SF Tuolumne R | CA | 37.8113 | 119.9367 | 2/7/2016 |
| *Arsapnia utahensis* | 132 | M | X | X | X | X | South Cr | CA | 35.9693 | 118.4878 | 2/6/2016 |
| *Arsapnia utahensis* | 133 | F | X | X | X | X | South Cr | CA | 35.9693 | 118.4878 | 2/6/2016 |
| *Arsapnia utahensis* | 134 | M | X | X | X | X | Brush Cr | CA | 35.9660 | 118.4781 | 2/6/2016 |
| *Arsapnia utahensis* | 135 | F | X |  |  |  | Brush Cr | CA | 35.9660 | 118.4781 | 2/6/2016 |
| *Arsapnia utahensis* | 136 | M | X |  |  |  | Kern R | CA | 35.9700 | 118.4861 | 2/6/2016 |
| *Arsapnia utahensis* | 137 | M | X |  |  |  | Kern R | CA | 35.9700 | 118.4861 | 2/6/2016 |
| *Arsapnia utahensis* | 138 | F | X | X | X | X | Kern R | CA | 35.9700 | 118.4861 | 2/6/2016 |
| *Arsapnia utahensis* | 139 | F | X |  |  |  | Kern R | CA | 35.9700 | 118.4861 | 2/6/2016 |
| *Capnia californica* | 140 | M | X | X | X | X | E Verde R | AZ | 34.1805 | 111.2149 | 2/3/2016 |
| *Capnia umpqua* | 141 | M | X | nd | X | X | Mill Cr | CA | 39.3560 | 122.6551 | 2/8/2016 |
| *Capnia umpqua* | 142 | F | X | nd | X | X | Mill Cr | CA | 39.3560 | 122.6551 | 2/8/2016 |
| *Capnia spinulosa* | 143 | M | X | nd | nd | X | Fulton Cr | CA | 35.7226 | 118.6785 | 2/6/2016 |
| *Capnia spinulosa* | 144 | F | X | X | X | X | Fulton Cr | CA | 35.7226 | 118.6785 | 2/6/2016 |
| *Capnia kersti* | 145 | M | X | nd | X |  | Trib to Spencer Cr | OR | 43.9599 | 123.2077 | 2/10/2016 |
| *Capnia kersti* | 146 | F | X |  |  | X | Trib to Spencer Cr | OR | 43.9599 | 123.2077 | 2/10/2016 |
| *Capnia californica* | 147 | M | X | nd | X |  | Trib to SF Stony Cr | CA | 39.3645 | 122.6557 | 2/8/2016 |
| *Capnia californica* | 148 | M | X |  |  | X | Trib to SF Stony Cr | CA | 39.3645 | 122.6557 | 2/8/2016 |
| *Bolshecapnia maculata* | 149 | M | X | X | nd | X | Trib to SF Stony Cr | CA | 39.3645 | 122.6557 | 2/8/2016 |
| *Bolshecapnia maculata* | 150 | F | X |  | nd | X | Trib to SF Stony Cr | CA | 39.3645 | 122.6557 | 2/8/2016 |
| *Arsapnia tumida* | 151 | M | X | X | X | X |  | CA |  |  |  |
| *Capnura wanica* | 152 | M | X |  |  |  | Elkhorn Cr | CO | 40.6978 | 105.4408 | 3/21/2013 |
| *Capnura wanica* | 153 | F | X |  |  | X | Elkhorn Cr | CO | 40.6978 | 105.4408 | 3/21/2013 |
| *Capnura wanica* | 154 | M | X |  |  |  | Elkhorn Cr | CO | 40.6978 | 105.4408 | 3/18/2013 |
| *Capnura wanica* | 155 | F | X | X | X | X | Elkhorn Cr | CO | 40.6978 | 105.4408 | 3/18/2013 |
| *Utacapnia poda* | 156 | M | X | nd | X | X | Fraser R | CO | 40.0813 | 105.9306 | 3/17/2014 |
| *Utacapnia poda* | 157 | M | X | X | X | X | Fraser R | CO | 40.0813 | 105.9306 | 3/17/2014 |
| *Utacapnia poda* | 158 | F | X | X | X | X | Fraser R | CO | 40.0813 | 105.9306 | 3/17/2014 |
| *Capnia confusa* | 159 | M | X | nd | X | X | Elkhorn Cr | CO | 40.6978 | 105.4408 | 5/3/2013 |
| *Capnura wanica* | 160 | M | X |  |  |  | Elkhorn Cr | CO | 40.6978 | 105.4408 | 3/28/2013 |
| *Capnura wanica* | 161 | M | X | nd | X | X | Elkhorn Cr | CO | 40.6978 | 105.4408 | 3/28/2013 |
| *Capnia uintahi* | 162 | M | X | nd | X |  | S Cow Cr | CO | 39.8776 | 106.2724 | 3/17/2014 |
| *Capnia uintahi* | 163 | M | X | nd | X |  | S Cow Cr | CO | 39.8776 | 106.2724 | 3/17/2014 |
| *Capnia uintahi* | 164 | F | X |  |  | X | S Cow Cr | CO | 39.8776 | 106.2724 | 3/17/2014 |
| *Capnia uintahi* | 165 | F | X |  |  | X | S Cow Cr | CO | 39.8776 | 106.2724 | 3/17/2014 |
| *Capnia confusa* | 170 | M | X |  |  |  | SF White R | CO | 39.8648 | 107.5362 | 4/11/2015 |
| *Capnia confusa* | 171 | M | X | nd | X | X | SF White R | CO | 39.8648 | 107.5362 | 4/11/2015 |
| *Capnia confusa* | 172 | F | X |  |  |  | SF White R | CO | 39.8648 | 107.5362 | 4/11/2015 |
| *Capnia confusa* | 173 | F | X | nd | X | X | SF White R | CO | 39.8648 | 107.5362 | 4/11/2015 |
| *Arsapnia arapahoe* | 174 | M | H01 | X | X | X | Fourmile Cr | CO | 40.0363 | 105.3504 | 2/25/2016 |
| *Paracapnia baumanni* | 175 | M | X | X | X | X | Trib to Canyon Cr | CA | 40.7737 | 123.0506 | 2/23/2016 |
| *Paracapnia baumanni* | 176 | M | X |  |  |  | Trib to Canyon Cr | CA | 40.7737 | 123.0506 | 2/23/2016 |
| *Arsapnia pileata* | 177 | M | H21 | X | X | X | Fourmile Cr | CA | 40.8881 | 123.6434 | 2/23/2016 |
| *Arsapnia pileata* | 178 | F | H21 |  |  |  | Fourmile Cr | CA | 40.8881 | 123.6434 | 2/23/2016 |
| *Arsapnia pileata* | 179 | F | X |  |  |  | Fourmile Cr | CA | 40.8881 | 123.6434 | 2/23/2016 |
| *Arsapnia arapahoe* | 180 | M | H04 |  |  | X | Lost Gul | CO | 40.0073 | 105.3245 | 3/29/2016 |
| *Arsapnia arapahoe* | 181 | M | H04 |  |  |  | Young Gul | CO | 40.6893 | 105.3495 | 2/17/2016 |
| *Arsapnia coyote* | 184 | M | H18 | nd | X | X | EF WF Mojave R | CA | 34.2707 | 117.2946 | 1/9/2012 |
| *Arsapnia arapahoe* | 188 | M | H01 |  |  | X | Boulder Cr | CO | 40.0145 | 105.3112 | 3/6/2016 |
| *Arsapnia arapahoe* | 189 | M | H04 |  |  | X | Tom Davis Gul | CO | 39.9430 | 105.3366 | 3/4/2016 |
| *Arsapnia arapahoe* | 190 | M | H01 |  |  | X | Tom Davis Gul | CO | 39.9430 | 105.3366 | 3/4/2016 |
| *Arsapnia decepta* | 191 | M | H01 | X | X |  | Central Gul | CO | 40.1750 | 105.3440 | 4/26/2013 |
| *Arsapnia decepta* | 192 | M | H01 |  |  |  | Central Gul | CO | 40.1750 | 105.3440 | 4/26/2013 |
| *Arsapnia decepta* | 193 | F | H01 |  |  |  | Central Gul | CO | 40.1750 | 105.3440 | 4/26/2013 |
| *Arsapnia decepta* | 194 | F | H01 |  |  |  | Central Gul | CO | 40.1750 | 105.3440 | 4/26/2013 |
| *Arsapnia decepta* | 195 | M | H02 | nd | X | X | Boulder Cr | CO | 40.0131 | 105.3013 | 3/16/2014 |
| *Arsapnia decepta* | 196 | M | H04 | X | X |  | Boulder Cr | CO | 40.0131 | 105.3013 | 3/16/2014 |
| *Arsapnia decepta* | 197 | F | H04 |  |  | X | Boulder Cr | CO | 40.0131 | 105.3013 | 3/16/2014 |
| *Arsapnia decepta* | 198 | F | H04 |  |  | X | Boulder Cr | CO | 40.0131 | 105.3013 | 3/16/2014 |
| *Arsapnia decepta* | 199 | M | H01* |  |  |  | Eightmile Cr | CO | 38.5449 | 105.1097 | 3/7/2014 |
| *Arsapnia decepta* | 200 | M | H01* |  |  |  | Eightmile Cr | CO | 38.5449 | 105.1097 | 3/7/2014 |
| *Arsapnia decepta* | 201 | F | H06 | nd | X | X | Eightmile Cr | CO | 38.5449 | 105.1097 | 3/7/2014 |
| *Arsapnia decepta* | 202 | F | H01 | X | X |  | Eightmile Cr | CO | 38.5449 | 105.1097 | 3/7/2014 |
| *Arsapnia decepta* | 203 | M | H01 |  |  |  | Greenhorn Cr | CO | 37.9205 | 104.9561 | 3/29/2013 |
| *Arsapnia decepta* | 204 | F | H05 | X | X | X | Greenhorn Cr | CO | 37.9205 | 104.9561 | 3/29/2013 |
| *Arsapnia decepta* | 205 | F | X |  |  |  | Greenhorn Cr | CO | 37.9205 | 104.9561 | 3/29/2013 |
| *Arsapnia decepta* | 206 | M | H01 |  |  |  | Sheep Cr | CO | 40.5708 | 105.3483 | 3/14/2013 |
| *Arsapnia decepta* | 207 | M | H01 |  |  |  | Sheep Cr | CO | 40.5708 | 105.3483 | 3/14/2013 |
| *Arsapnia decepta* | 208 | F | H04 |  |  |  | Sheep Cr | CO | 40.5708 | 105.3483 | 3/14/2013 |
| *Arsapnia decepta* | 209 | F | H04 | nd | X | X | Sheep Cr | CO | 40.5708 | 105.3483 | 3/14/2013 |
| *Arsapnia decepta* | 210 | M | H01 | nd | X |  | Sheep Cr | CO | 40.5708 | 105.3483 | 5/11/2013 |
| *Arsapnia decepta* | 211 | M | H04 |  |  |  | Sheep Cr | CO | 40.5708 | 105.3483 | 5/11/2013 |
| *Arsapnia decepta* | 212 | F | H01 |  |  |  | Sheep Cr | CO | 40.5708 | 105.3483 | 5/11/2013 |
| *Arsapnia decepta* | 213 | F | H03 | nd | X | X | Sheep Cr | CO | 40.5708 | 105.3483 | 5/11/2013 |
| *Arsapnia decepta* | 214 | M | H04 | X | X |  | Van Bibber Cr | CO | 39.8014 | 105.2499 | 2/10/2015 |
| *Arsapnia decepta* | 215 | M | H01 |  |  |  | Van Bibber Cr | CO | 39.8014 | 105.2499 | 2/10/2015 |
| *Arsapnia decepta* | 216 | F | H04 |  |  |  | Van Bibber Cr | CO | 39.8014 | 105.2499 | 2/10/2015 |
| *Arsapnia decepta* | 217 | F | H01 |  |  |  | Van Bibber Cr | CO | 39.8014 | 105.2499 | 2/10/2015 |
| *Arsapnia arapahoe* | 220 | M | H01 | nd | X | X | Bear Canyon Cr | CO | 39.9753 | 105.2817 | 2/16/2014 |
| *Arsapnia arapahoe* | 221 | M | H01 |  |  | X | Fourmile Cr | CO | 40.0616 | 105.2893 | 3/9/2015 |
| *Arsapnia arapahoe* | 222 | F | X |  |  |  | Fourmile Cr | CO | 40.0616 | 105.2893 | 3/9/2015 |
| *Arsapnia arapahoe* | 223 | M | H01 |  |  | X | Elkhorn Cr | CO | 40.6980 | 105.4404 | 3/6/2009 |
| *Arsapnia arapahoe* | 224 | M | H04 |  |  |  | Elkhorn Cr | CO | 40.6980 | 105.4404 | 3/6/2009 |
| *Arsapnia arapahoe* | 225 | F | X |  |  |  | Elkhorn Cr | CO | 40.6980 | 105.4404 | 3/6/2009 |
| *Arsapnia arapahoe* | 226 | M | H04 |  |  |  | Bear Canyon Cr | CO | 39.9753 | 105.2817 | 1/30/2015 |
| *Arsapnia arapahoe* | 227 | M | H01 |  |  | X | Martin Gul | CO | 39.9463 | 105.3156 | 2/27/2014 |
| *Arsapnia arapahoe* | 228 | M | H04 | X | X |  | Martin Gul | CO | 39.9463 | 105.3156 | 2/27/2014 |
| *Arsapnia arapahoe* | 229 | M | H01 | nd | X |  | Elkhorn Cr | CO | 40.6980 | 105.4404 | 3/21/2013 |
| *Arsapnia arapahoe* | 230 | M | H01 |  |  |  | Elkhorn Cr | CO | 40.6978 | 105.4408 | 4/9/2014 |
| *Arsapnia arapahoe* | 231 | M | H01 |  |  |  | Tom Davis Gul | CO | 39.9434 | 105.3372 | 3/14/2014 |
| *Arsapnia arapahoe* | 232 | M | nd |  |  | X | Elkhorn Cr | CO | 40.6980 | 105.4404 | 3/6/2009 |
| *Arsapnia arapahoe* | 233 | M | H01 |  |  | X | Bummers Gul | CO | 40.0187 | 105.3684 | 2/28/2014 |
| *Capnia gracilaria* | 236 | M |  |  |  | X | Tom Davis Gul | CO | 39.9453 | 105.3406 | 3/14/2014 |
| *Capnia gracilaria* | 241 | M |  |  |  | X | Tom Davis Gul | CO | 39.9453 | 105.3406 | 3/14/2014 |
| *Capnia gracilaria* | 242 | M |  |  |  | X | Tom Davis Gul | CO | 39.9453 | 105.3406 | 3/14/2014 |
| *Capnia gracilaria* | 243 | M |  |  |  | X | Tom Davis Gul | CO | 39.9453 | 105.3406 | 3/14/2014 |
| *Capnia gracilaria* | 244 | M |  |  |  | X | Tom Davis Gul | CO | 39.9453 | 105.3406 | 3/14/2014 |
| *Arsapnia decepta* | 253 | M |  |  |  | X | Tom Davis Gul | CO | 39.9453 | 105.3406 | 3/4/2016 |
| *Arsapnia decepta* | 254 | M |  |  |  | X | Tom Davis Gul | CO | 39.9453 | 105.3406 | 3/4/2016 |
| *Arsapnia decepta* | 255 | M |  |  |  | X | Tom Davis Gul | CO | 39.9453 | 105.3406 | 3/4/2016 |
| *Arsapnia decepta* | 256 | M |  |  |  | X | Tom Davis Gul | CO | 39.9453 | 105.3406 | 3/4/2016 |
| *Arsapnia decepta* | 262 | M |  |  |  | X | Tom Davis Gul | CO | 39.9453 | 105.3406 | 3/4/2016 |
| *Arsapnia decepta* | 263 | M |  |  |  | X | Tom Davis Gul | CO | 39.9453 | 105.3406 | 3/4/2016 |
| *Arsapnia decepta* | 264 | M |  |  |  | X | Tom Davis Gul | CO | 39.9453 | 105.3406 | 3/4/2016 |
| *Arsapnia decepta* | 265 | M |  |  |  | X | Tom Davis Gul | CO | 39.9453 | 105.3406 | 3/4/2016 |
| *Arsapnia decepta* | 271 | M |  |  |  | X | Elkhorn Cr | CO | 40.6978 | 105.4408 | 4/20/2013 |
| *Arsapnia decepta* | 276 | F |  |  |  | X | Elkhorn Cr | CO | 40.6978 | 105.4408 | 4/20/2013 |
| *Arsapnia decepta* | 277 | F |  |  |  | X | Elkhorn Cr | CO | 40.6978 | 105.4408 | 4/20/2013 |
| *Arsapnia decepta* | 278 | F |  |  |  | X | Elkhorn Cr | CO | 40.6978 | 105.4408 | 4/20/2013 |
| *Arsapnia decepta* | 279 | F | H01 |  |  | X | Elkhorn Cr | CO | 40.6978 | 105.4408 | 4/20/2013 |
| *Arsapnia decepta* | 281 | F |  |  |  | X | Elkhorn Cr | CO | 40.6978 | 105.4408 | 4/20/2013 |
| *Arsapnia decepta* | 286 | F |  |  |  | X | Bummers Gul | CO | 40.0187 | 105.3684 | 2/22/2014 |
| *Arsapnia decepta* | 287 | F |  |  |  | X | Bummers Gul | CO | 40.0187 | 105.3684 | 2/22/2014 |
| *Arsapnia decepta* | 288 | M |  |  |  | X | Bummers Gul | CO | 40.0187 | 105.3684 | 2/22/2014 |
| *Arsapnia decepta* | 296 | F |  |  |  | X | Bummers Gul | CO | 40.0187 | 105.3684 | 2/22/2014 |
| *Arsapnia decepta* | 297 | M |  |  |  | X | Bummers Gul | CO | 40.0187 | 105.3684 | 2/22/2014 |
| *Arsapnia decepta* | 298 | M |  |  |  | X | Bummers Gul | CO | 40.0187 | 105.3684 | 2/22/2014 |
| *Arsapnia decepta* | 314 | F |  |  |  | X | Boulder Cr | CO | 40.0131 | 105.3013 | 3/16/2014 |
| *Arsapnia decepta* | 315 | F |  |  |  | X | Boulder Cr | CO | 40.0131 | 105.3013 | 3/16/2014 |
| *Arsapnia decepta* | 318 | M |  |  |  | X | Boulder Cr | CO | 40.0131 | 105.3013 | 3/16/2014 |
| *Arsapnia decepta* | 321 | F |  |  |  | X | Boulder Cr | CO | 40.0131 | 105.3013 | 3/16/2014 |
| *Arsapnia decepta* | 323 | F |  |  |  | X | Sheep Cr | CO | 40.5708 | 105.3483 | 3/14/2013 |
| *Arsapnia decepta* | 324 | F |  |  |  | X | Sheep Cr | CO | 40.5708 | 105.3483 | 3/14/2013 |
| *Arsapnia decepta* | 328 | F |  |  |  | X | Sheep Cr | CO | 40.5708 | 105.3483 | 3/14/2013 |
| *Arsapnia decepta* | 330 | F |  |  |  | X | Sheep Cr | CO | 40.5708 | 105.3483 | 3/14/2013 |
| *Arsapnia decepta* | 332 | M |  |  |  | X | Sheep Cr | CO | 40.5708 | 105.3483 | 3/14/2013 |
| *Arsapnia decepta* | 333 | M |  |  |  | X | Sheep Cr | CO | 40.5708 | 105.3483 | 3/14/2013 |
| *Capnia gracilaria* | 334 | M |  |  |  | X | Bummers Gul | CO | 40.0183 | 105.3675 | 2/28/2014 |
| *Capnia gracilaria* | 335 | M |  |  |  | X | Bummers Gul | CO | 40.0183 | 105.3675 | 2/28/2014 |
| *Capnia gracilaria* | 337 | M |  |  |  | X | Bummers Gul | CO | 40.0183 | 105.3675 | 2/28/2014 |
| *Capnia gracilaria* | 338 | M |  |  |  | X | Bummers Gul | CO | 40.0183 | 105.3675 | 2/28/2014 |
| *Capnia gracilaria* | 340 | M |  |  |  | X | Bummers Gul | CO | 40.0183 | 105.3675 | 2/28/2014 |
| *Capnia gracilaria* | 343 | M |  |  |  | X | Elkhorn Cr | CO | 40.6978 | 105.4408 | 3/28/2013 |
| *Capnia gracilaria* | 345 | F |  |  |  | X | Elkhorn Cr | CO | 40.6978 | 105.4408 | 3/28/2013 |
| *Capnia gracilaria* | 346 | F |  |  |  | X | Elkhorn Cr | CO | 40.6978 | 105.4408 | 3/28/2013 |
| *Capnia gracilaria* | 347 | F |  |  |  | X | Elkhorn Cr | CO | 40.6978 | 105.4408 | 3/28/2013 |
| *Capnia gracilaria* | 348 | F |  |  |  | X | Elkhorn Cr | CO | 40.6978 | 105.4408 | 3/28/2013 |
| *Capnura wanica* | 357 | M |  |  |  | X | Elkhorn Cr | CO | 40.6978 | 105.4408 | 3/28/2013 |
| *Capnura wanica* | 358 | F |  |  |  | X | Elkhorn Cr | CO | 40.6978 | 105.4408 | 3/28/2013 |
| *Capnura wanica* | 359 | M |  |  |  | X | Sheep Cr | CO | 40.5706 | 105.3483 | 3/14/2013 |

^1^Code is the laboratory code. Interruptions in the sequence of codes reflect specimens for which all genetic analyses failed.

^2^Sex: male (M), female (F).

^3^Genetic data: X, sequenced; H01–H22, haplotype labels for the *Arsapnia* group in Figure 2. An asterisk denotes a sequence with missing data, but which is otherwise identical to a particular haplotype; nd, sequencing or amplification failed.

Table S2. Sequences of specimens from public databases.

| Gene | BOLD | GenBank | Taxon^1^ | Haplotype^2^ |
| --- | --- | --- | --- | --- |
| COI | CAD001-13 |  | *Arsapnia arapahoe* (M) | H01 |
|  | CAD002-13 |  | *Arsapnia arapahoe* (M) | H04 |
|  | CAD003-13 |  | *Arsapnia arapahoe* (M) | H04 |
|  | CAD004-13 |  | *Arsapnia arapahoe* (M) | H01 |
|  | CAD005-13 |  | *Arsapnia arapahoe* (M) | H01 |
|  | CAD006-13 |  | *Arsapnia arapahoe* (M) | H01 |
|  | CAD007-13 |  | *Arsapnia arapahoe* (M) | H01 |
|  | CAD008-13 |  | *Arsapnia arapahoe* (M) | H04 |
|  | CAD009-13 |  | *Arsapnia arapahoe* (M) | H04 |
|  | CAD010-13 |  | *Arsapnia arapahoe* (M) | H01 |
|  | CAD011-13 |  | *Arsapnia arapahoe* (M) | H01 |
|  | CAD012-13 |  | *Arsapnia arapahoe* (M) | H04 |
|  | CAD013-13 |  | *Arsapnia arapahoe* (M) | H01 |
|  | CAD024-13 |  | *Arsapnia arapahoe* (M) | 1 bp from H01, H04* |
|  | CAD025-13 |  | *Arsapnia arapahoe* (M) | H01 |
|  | CAD026-13 |  | *Arsapnia arapahoe* (M) | H04 |
|  | CAD027-13 |  | *Arsapnia arapahoe* (M) | H04 |
|  | CAD028-13 |  | *Arsapnia arapahoe* (M) | H04 |
|  | CSUCN001-13 |  | *Arsapnia arapahoe* (M) | H01, H04* |
|  | CAD031-14 |  | *Arsapnia coyote* (F) | H17* |
|  | CAD032-14 |  | *Arsapnia coyote* (F) | H12* |
|  | CAD033-14 |  | *Arsapnia coyote* (M) | H13 |
|  |  | HQ554575 | *Arsapnia decepta* | H04 |
|  |  | HQ554576 | *Arsapnia decepta* | H01 |
|  |  | HQ554577 | *Arsapnia decepta* | H01 |
|  | WEAI075-11 |  | *Arsapnia decepta* | H01 |
|  | INRMA633-12 |  | *Capnia coloradensis* |  |
|  | INRMA635-12 |  | *Capnia coloradensis* |  |
|  | INRMA636-12 |  | *Capnia coloradensis* |  |
|  |  | MG374808 | *Capnia coloradensis* |  |
|  |  | MG375669 | *Capnia coloradensis* |  |
|  |  | MG378228 | *Capnia coloradensis* |  |
|  |  | MG380637 | *Capnia coloradensis* |  |
|  |  | MG380731 | *Capnia coloradensis* |  |
|  |  | MG380949 | *Capnia coloradensis* |  |
|  |  | MG382570 | *Capnia coloradensis* |  |
|  |  | MG383283 | *Capnia coloradensis* |  |
|  | SMTPM6693-15 |  | *Capnia coloradensis* |  |
|  | SMTPM6799-15 |  | *Capnia coloradensis* |  |
|  | INRMA2034-15 |  | *Capnia coloradensis* (M) |  |
|  | BKSTO089-08 |  | *Capnia confusa* |  |
|  | BKSTO090-08 |  | *Capnia confusa* |  |
|  | BKSTO091-08 |  | *Capnia confusa* |  |
|  | BKSTO092-08 |  | *Capnia confusa* |  |
|  | BKSTO093-08 |  | *Capnia confusa* |  |
|  | BKSTO094-08 |  | *Capnia confusa* |  |
|  | BKSTO119-08 |  | *Capnia confusa* |  |
|  | BKSTO120-08 |  | *Capnia confusa* |  |
|  | BKSTO121-08 |  | *Capnia confusa* |  |
|  | BKSTO122-08 |  | *Capnia confusa* |  |
|  | BKSTO123-08 |  | *Capnia confusa* |  |
|  | BKSTO124-08 |  | *Capnia confusa* |  |
|  | INRMA572-12 |  | *Capnia confusa* |  |
|  |  | KM528390 | *Capnia confusa* |  |
|  |  | KM530900 | *Capnia confusa* |  |
|  |  | KM533911 | *Capnia confusa* |  |
|  | BKSTO032-08 |  | *Capnia excavata* |  |
|  | BKSTO033-08 |  | *Capnia excavata* |  |
|  | BKSTO034-08 |  | *Capnia excavata* |  |
|  | BKSTO434-11 |  | *Capnia glabra* |  |
|  | BKSTO416-11 |  | *Capnia gracilaria* |  |
|  | BKSTO417-11 |  | *Capnia gracilaria* |  |
|  | BKSTO418-11 |  | *Capnia gracilaria* |  |
|  | BKSTO419-11 |  | *Capnia gracilaria* |  |
|  | EVOTR153-12 |  | *Capnia gracilaria* |  |
|  | EVOTR154-12 |  | *Capnia gracilaria* |  |
|  | EVOTR155-12 |  | *Capnia gracilaria* |  |
|  | INRMA640-12 |  | *Capnia gracilaria* |  |
|  | INRMA641-12 |  | *Capnia gracilaria* |  |
|  | INRMA642-12 |  | *Capnia gracilaria* |  |
|  | INRMA643-12 |  | *Capnia gracilaria* |  |
|  | INRMA587-12 |  | *Capnia gracilaria* (M) |  |
|  | INRMA659-12 |  | *Capnia gracilaria* (M) |  |
|  | INRMA660-12 |  | *Capnia gracilaria* (M) |  |
|  | BKSTO437-11 |  | *Capnia melia* |  |
|  | BKSTO107-08 |  | *Capnia nana* |  |
|  | BKSTO108-08 |  | *Capnia nana* |  |
|  | BKSTO109-08 |  | *Capnia nana* |  |
|  | BKSTO110-08 |  | *Capnia nana* |  |
|  | BKSTO111-08 |  | *Capnia nana* |  |
|  | BKSTO112-08 |  | *Capnia nana* |  |
|  | INRMA2036-15 |  | *Capnia nana* |  |
|  | INRMA2037-15 |  | *Capnia nana* |  |
|  | INRMA637-12 |  | *Capnia nana* |  |
|  | INRMA638-12 |  | *Capnia nana* |  |
|  | INRMA2031-15 |  | *Capnia petila* |  |
|  | INRMA2032-15 |  | *Capnia petila* |  |
|  |  | KM530056 | *Capnia petila* |  |
|  |  | KR941840 | *Capnia petila* |  |
|  | INRMA583-12 |  | *Capnia petila* (F) |  |
|  | BKSTO401-11 |  | *Capnia vernalis* |  |
|  | BKSTO402-11 |  | *Capnia vernalis* |  |
|  | BKSTO403-11 |  | *Capnia vernalis* |  |
|  | BKSTO467-11 |  | *Capnia vernalis* |  |
|  | BKSTO468-11 |  | *Capnia vernalis* |  |
|  | BKSTO469-11 |  | *Capnia vernalis* |  |
|  |  | MG374347 | *Capnia vernalis* |  |
|  |  | MG375767 | *Capnia vernalis* |  |
|  |  | MG378194 | *Capnia vernalis* |  |
|  |  | MG380968 | *Capnia vernalis* |  |
|  |  | MG383077 | *Capnia vernalis* |  |
|  |  | MG383361 | *Capnia vernalis* |  |
|  | BKSTO444-11 |  | *Sierracapnia barberi* |  |
|  | BKSTO445-11 |  | *Sierracapnia barberi* |  |
|  |  |  |  |  |
|  |  |  |  |  |
| cyt *b* |  | KP642637 | *Mesocapnia arizonensis* |  |
|  |  | NC034661 | *Capnia zijinshana* |  |
|  |  |  |  |  |
| ITS1 |  | KF671096 | *Capnia bifrons* |  |
|  |  | KF671098 | *Capnia bifrons* |  |
|  |  | KF671100 | *Capnia bifrons* |  |

^1^Where provided, sex is noted in parentheses; male (M), female (F).

^2^Haplotypes from Figure 2. An asterisk denotes a sequence with missing data, but which is otherwise identical (or nearly so) to a particular haplotype.


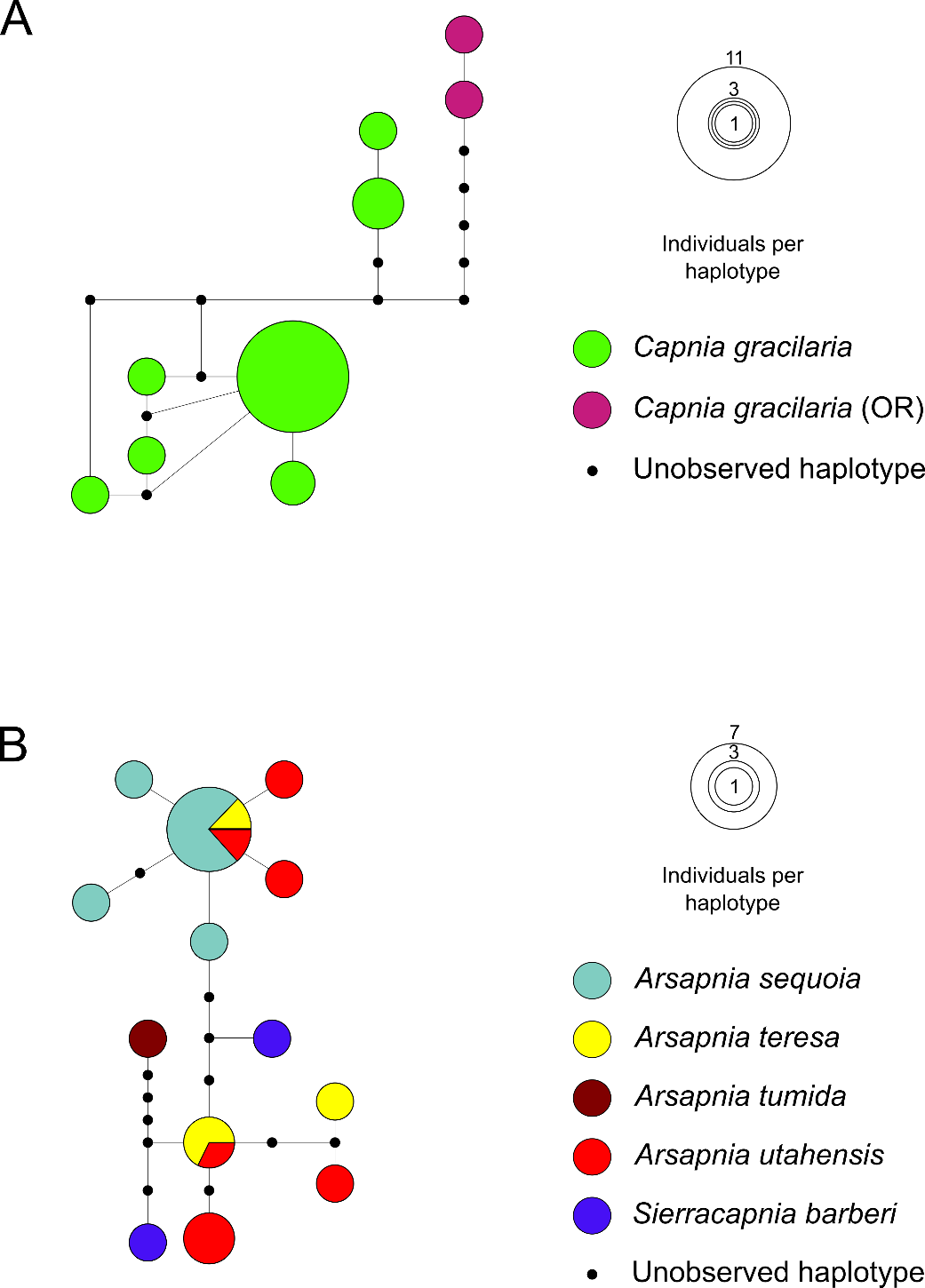


Figure S1**.** The two additional 95% maximum parsimony networks of cytochrome c oxidase subunit 1 sequences for haplotypes of capniid stoneflies from the *Arsapnia* group. Figure 1 depicts those haplotypes joined to the network containing *Arsapnia arapahoe*. Panel A, network containing haplotypes (*n* = 9) related to *Capnia gracilaria*. Panel B, network containing haplotypes (*n* = 13) of remaining members of the *Arsapnia* group. Each circle represents a haplotype, sizes are proportional to the number of individuals with that haplotype, and phenotypes associated with each haplotype are identified by color. Each line segment represents a single mutation, and small black dots represent unobserved haplotypes.

Figure S2. The best-scoring phylogenetic tree inferred from a data-partitioned maximum-likelihood analysis (with 1,000 bootstrap replicates) of 91 sequences (1,419 nucleotides) of the concatenated mitochondrial genes cytochrome c oxidase subunit 1 and cytochrome b. Sex of a specimen is indicated by M (male) or F (female). Bootstrap support values are given at branch nodes. For sequence numbers, see Table S1.
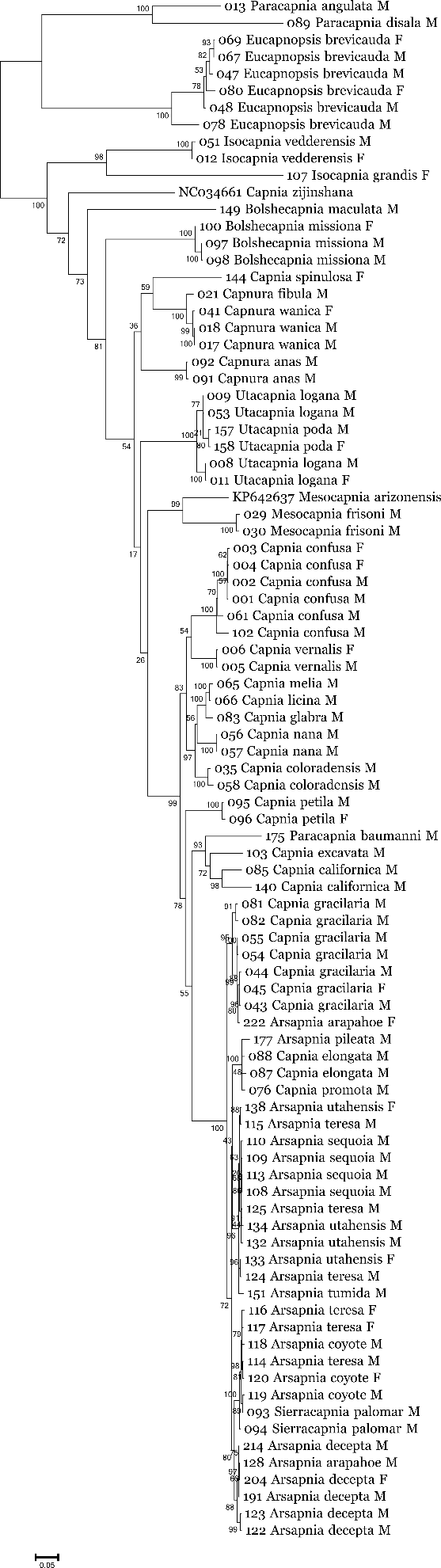


Figure 3. The best-scoring phylogenetic tree inferred from a data-partitioned maximum-likelihood analysis (with 1,000 bootstrap replicates) of 64 sequences (442 nucleotides and 87 gap-coded positions) of the nuclear first internal transcribed spacer and adjacent portions of the r18S and r5.8S regions. Sex of a specimen is indicated by M (male) or F (female). Bootstrap support values are given at branch nodes. For sequence numbers, see Table S1.
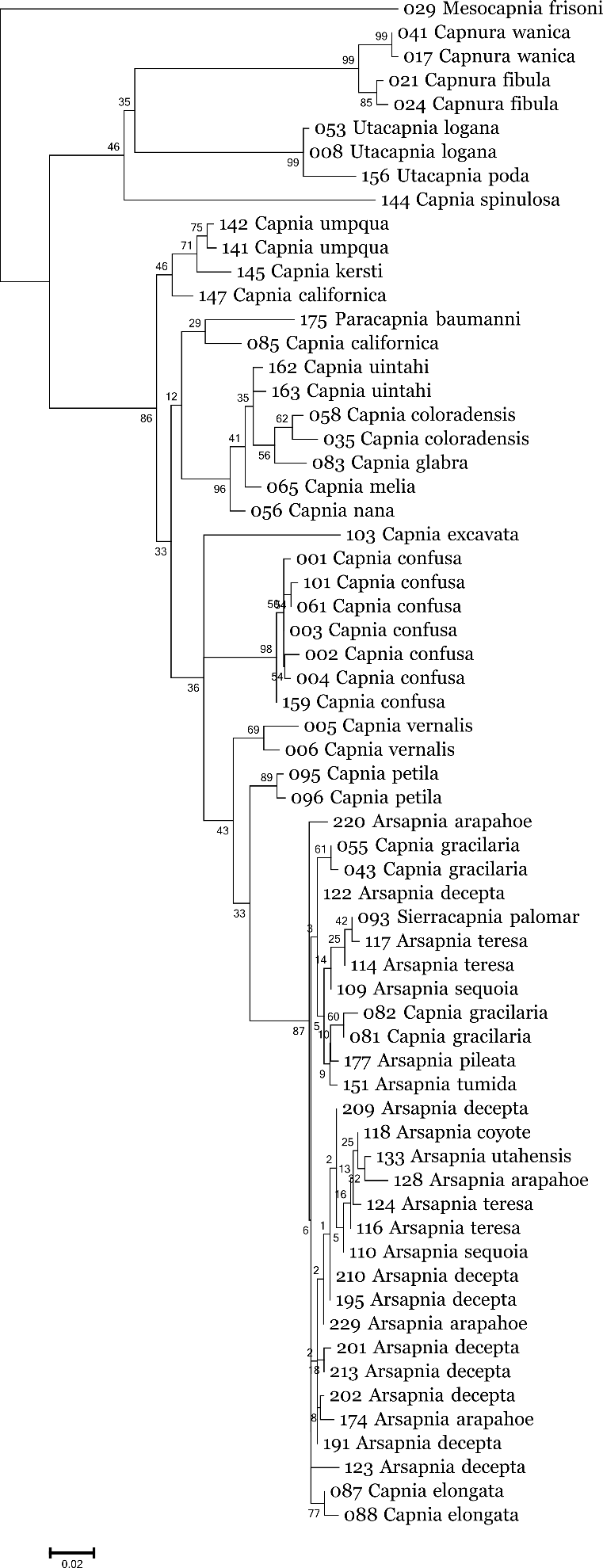

Supplement: Supplementary file 1 [file ECE3-9-1364-s001.docx]
